# Steady-state epithelial apical flatness is characterized by MLCK morphodynamics and asynchronous Ca^2+^ oscillations, but not by underlying ECM geometry

**DOI:** 10.1091/mbc.E25-12-0583

**Published:** 2026-04-14

**Authors:** Hao Wu, Emerson Herrmann, Jeanette Hyer, Lisa Hua, Takashi Mikawa

**Affiliations:** ^a^Cardiovascular Research Institute, University of California San Francisco 555 Mission Bay Blvd South, San Francisco, CA 94158; ^b^Department of Biology, Sonoma State University 1801 East Cotati Ave Rohnert Park, CA 94928; Duke University

## Abstract

The canonical simple epithelium is a flat sheet-like tissue of horizontally packed cells. While the basal surface is delineated by the basement membrane of extracellular matrix (ECM), little is known about how a flat apical surface is maintained, or if apical/basal dynamics are coordinated. The current study tests the role of the apical domain to define mechanisms involved in maintaining a flat apical geometry in an epithelium. When the basal geometry is modulated, Madin-Darby Canine Kidney (MDCK) cells adjust their morphology to maintain an overall apical flatness of the confluent layer. Pharmacological and transgenic disruption of non-muscle myosin ATPase and MLCK activity results in an uneven apical structure and overall loss of the flat geometry typical of a confluent epithelium. Surprisingly, transgenic experimentation showed that forces maintaining individual MDCK cell flatness are cell-autonomous. Finally, Ca^2+^ imaging reveals an asynchronous calcium flux across a confluent epithelium. Our results highlight that apical/basal cellular surfaces may not be tightly coordinated, but rather independently regulated. This study provides a new paradigm for how apical flatness is regulated at steady state.

## Abstract (executive summary)

- Simple epithelial cells form monolayers with a flat, uniform apical surface. How the flatness of a steady-state epithelium is established and maintained is unknown.
- Modifications to the ECM structure, pharmacological and molecular perturbation applied to MDCK cells were used to examine changes in monolayer flatness. The data revealed that apical flatness is controlled independently from basement membrane geometry and requires balancing of myosin II morphodynamics.
- As steady-state epithelial tissue function depends largely on epithelial cell structure and integrity, it is essential to understand the underlying mechanisms that maintain this cell morphology. Loss of this formation can be implicated in cancer.

## INTRODUCTION

Simple epithelia sit on a basement membrane of extracellular matrix (ECM; Martin-Belmonte and Mostov, 2008; Kozyrina *et al.*, 2020; Khalilgharibi and Mao, 2021). At the tissue level, epithelial cells assemble to form a steady-state epithelium, characterized by the balance of cell proliferation and death to maintain a flat, unified apical surface resistant to external stressors (Odenwald *et al.*, 2018; Tai *et al.*, 2019; Lemke and Nelson, 2021; Banjac *et al.*, 2023) Loss of this formation has been implicated in different types of cancer (Coradini *et al.*, 2011; Eisenhoffer and Rosenblatt, 2012; Hannezo *et al.*, 2014; Rodriguez-Boulan and Macara, 2014; Janiszewska *et al.*, 2020; Ribatti *et al.*, 2020; Peglion and Etienne-Manneville, 2024). To date, it is unclear how a uniform flat apical surface is maintained in a steady-state epithelium. In this study, we investigated the underlying mechanism(s) maintaining the flatness of the apical surface and its relationship with the geometry of the underlying basement membrane in a steady-state epithelium.

An actomyosin network exists at the lumen/cytoplasmic face of the adherens junctions, or apical side, of epithelial cells (Meng and Takeichi, 2009; Ebrahim *et al.*, 2013; Klingner *et al.*, 2014; Marivin *et al.*, 2022). In non-muscle cells, including epithelial cells, it is assumed that intracellular Ca^2+^ ion (iCa^2+^)-dependent phosphorylation of Myosin Regulatory Light Chain (MRLCs) generates mechanical force as seen in smooth muscle cells (Hathaway and Adelstein, 1979; Garrido-Casado *et al.*, 2024; Turner *et al.*, 2024). The stimulation of contractile force is released by opposing MRLC phosphatases (Bresnick, 1999; Watanabe *et al.*, 2007). Both myosin and its upstream regulators, Rho-kinase, Coiled-coil Kinase (ROCK), Myosin Light Chain Kinase (MLCK), and Myosin Light Chain Phosphatase (MLCP) are required for proper actomyosin contraction dynamics (Totsukawa *et al.*, 2000; Kassianidou *et al.*, 2017). Changes to the actomyosin contractile network have been shown to change epithelial cell morphology; in particular, this network can be used to drive apical constriction and tissue morphogenesis (Martin *et al.*, 2009; Martin and Goldstein, 2014; Chugh and Paluch, 2018; Clarke and Martin, 2021; Lemke and Nelson, 2021; Ranie and White, 2025). It is also unknown whether iCa^2+^-dependent phosphorylation of MRLCs is required for the regulation of a flat apical surface of a steady-state epithelium.

Using Madin-Darby Canine Kidney (MDCK) cell monolayers, the present study experimentally probed the mechanisms underlying the flatness of the apical epithelial surface, distinctly from the overall epithelial flatness. Here, we show that the flatness of an epithelial sheet was preserved after growth on irregular non-uniform basement matrices. We also reveal that pharmacological and molecular perturbations disrupting myosin activation caused a loss of apical surface flatness that was more severe than perturbations of the actin networks. Finally, we show that epithelial cells transiently and asynchronously evoked iCa^2+^ fluxes across the sheet, implying that calcium is available for a potential activation of MLCK-mediated mechanical force. Our data reveal that apical and basal cellular surfaces are not tightly coordinated, but independently regulated, and hint that myosin may have a role in the apical morphology of epithelial cells.

## RESULTS

### Definition of flatness using confluent MDCK monolayers

To evaluate epithelial architecture, we used MDCK cells, an established model for epithelial sheet formation (Misfeldt *et al.*, 1976; Dukes *et al.*, 2011; Wells *et al.*, 2013), and immunofluorescence labeling of polarity markers. Individual cell and apical height were quantified at both the tissue-level (macro-height) and cellular-level (micro-height) for MDCK confluent monolayers to define flatness. To assess the loss of flatness, three parameters were considered: 1) height differences between immediately adjacent cells, 2) the overall distribution of cell heights within a defined area, and 3) apical domain height of individual cells. These metrics were applied for macro- and micro-height measurements to characterize surface irregularity, or loss of apical flatness. In cases where the distributional analysis (parameter 2) and apical domain height (parameter 3) sufficiently capture variation in height and spatial heterogeneity, the pairwise height difference data (parameter 1) were omitted without compromising the evaluation of flatness.

**FIGURE 1:**
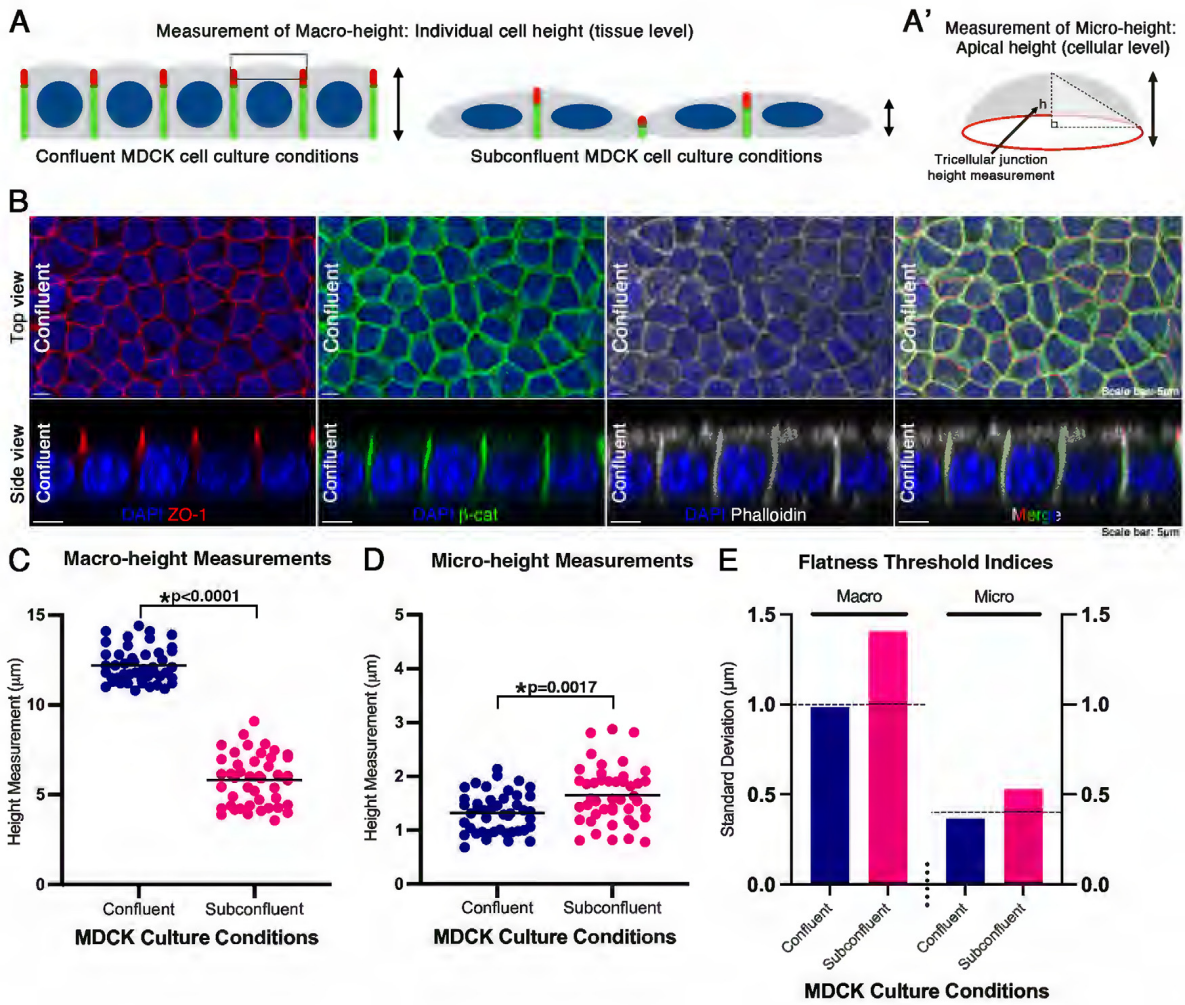
Definition of flatness using confluent MDCK monolayers. (A) Schematic showing the measurements of macro-height of individual MDCK cells when grown in confluent and subconfluent cell culture conditions. (A’) Micro-height or apical cell height was measured from the apex of filamentous actin staining to the base of the tricellular junctions. (B) Top and side views of stacked confocal optical sections of an MDCK confluent monolayer immunostained with ZO-1 (red), β-catenin (green), with filamentous actin dye, phalloidin (grey), and a DNA counterstain, DAPI (blue). (C) Average cell height or macro-height measurements for confluent MDCK cells were 12.21 µm ± 0.99 µm s.d., and 5.81 µm ± 1.40 µm s.d. for subconfluent cells (U = 0.00, n_1_ = 45, n_2_ = 45, *p* < 0.0001). (D) Same as (C) but of apical height or micro-height: 1.32 µm ± 0.37 µm s.d. for confluent cells, and 1.65 µm ± 0.53 µm s.d. for subconfluent cells (U = 628, n_1_ = 45, n_2_ = 45, *p* = 0.0017). Conditions were statistically compared with a nonparametric Mann-Whitney U test. Unequal variance was confirmed with Levene's test (*p* = 0.0093 for macro-height, *p* = 0.0274 for micro-height). (E) Standard deviations of macro- and micro-height of confluent and subconfluent monolayers. Flatness threshold indices are indicated with horizontal dashed lines. (Scale bars: 5µm)

Immunofluorescence for β-catenin and ZO-1 was used to define lateral and apical junctions, respectively, and phalloidin to define cell boundaries for measuring macro- and micro-height (Figure 1, A and A’). Our confocal 3D reconstruction analysis revealed that confluent MDCK cells showed stereotypical accumulation patterns of ZO-1, β-catenin, and phalloidin-bound actin bundles (Figure 1B), consistent with previous studies (McNeil *et al.*, 2006; Fanning *et al.*, 2012; Odenwald *et al.*, 2018; Leng *et al.*, 2020; Kuo *et al.*, 2022).

Trans-epithelial electrical resistance (TEER) measurements of MDCK cells were used to determine when confluent monolayers formed (Srinivasan *et al.*, 2015; Schimetz *et al.*, 2024). MDCK monolayers displayed the highest electrical resistance of 279.6 Ω•cm^2^ ± 33.23 Ω•cm^2^ SD (s.d.) at 45 h over a 3-d measurement period of the same monolayer (Supplemental Figure S1A), similar to other reported TEER measurements for MDCK cells (Fedi *et al.*, 2021). The data demonstrate that in our hands, 45 h was necessary to define a confluent monolayer on which we could complete our analysis (Supplemental Figure S1A).

Our data indicate that TEER and epithelial flatness are closely correlated during the approach to peak confluency. At 45–50 h, when TEER reaches its maximum, cells exhibit minimal variability in both macro- and micro-height measurements and display a uniform flat apical surface (Supplemental Figure S1, A, B, F–H). This suggests that the maximal barrier integrity coincides with maximal epithelial flattening and structural homogeneity of the monolayer (Chmiel and Gardel, 2022; Cammarota *et al.*, 2024). However, beyond peak confluence, the relationship becomes more complex. At 60–75 h, TEER declines to levels comparable to subconfluent MDCK cultures (Supplemental Figure S1, A and B). Despite this drop in barrier function, macro- and micro-height measurements at these later time points, defined as the “overconfluent monolayer”, remain closer to those of the confluent monolayer than to subconfluent cells (Supplemental Figure S1, E–H).

While we observe a decrease in macro-height in the overconfluent layer, micro-height remains mostly unchanged as compared with confluent layers (Supplemental Figure S1, F and G). However, there is a slight increase in macro-height variability, suggesting a slight loss of epithelial flatness in overconfluent monolayers (Supplemental Figure S1, F–H). Although the overall macro-height of the overconfluent monolayer is reduced, it does not revert to the variable, shorter morphology characteristic of subconfluent cells; instead, it displays an intermediate phenotype (Supplemental Figure S1, C–H). These findings suggest that TEER and epithelial flatness may be correlated during the establishment of the monolayer, but become partially uncoupled in overconfluent conditions.

Macro-height data were collected as a linear measure of the cell height from the basement membrane to the ZO-1 junctions (Figure 1A; Supplemental Figure S2, A and B). Micro-height was determined by measuring the degree of height difference between the midpoint of the phalloidin-positive apical surface and ZO-1-positive tricellular junctions (Figure 1A’; Supplemental Figure S2, A’-B). The average macro-height and micro-height measurements for the confluent MDCK monolayer were 12.21 µm ± 0.99 µm s.d. and 1.32 µm ± 0.37 µm s.d., respectively (*n* = 45 cells) (Figure 1C, D). The SD was used to generate a macro-height and micro-height index, and served as a threshold to evaluate if overall flatness was lost (Figure 1E).

To validate the threshold indices, we used subconfluent MDCK cells to confirm the expected morphology and reflect a loss of flatness. Subconfluent MDCK cells remain scattered and do not yet form a cohesive monolayer, providing a control for testing the validity of the threshold indices (Dawney *et al.*, 2023; Supplemental Figure S1D). Subconfluent MDCK cells displayed a higher degree of variation in both macro- and micro-height as compared with confluent monolayers (Figure 1, B–E; Supplemental Figure S1D). For example, the s.d. of macro-height for subconfluent cells was 1.40 µm and exceeded the threshold of a confluent monolayer, 0.99 µm s.d. (Figure 1, C and E). The s.d. of micro-height for subconfluent cells was 0.53 µm s.d., and also exceeded the threshold of confluent monolayers, 0.37 µm s.d. (*n* = 45 cells; Figure 1, D and E). Confluent monolayers and subconfluent cells were statistically compared with a Mann-Whitney U-test (*p* < 0.0001 for macro-height and *p* = 0.0017 for micro-height), and unequal variance was confirmed with Levene's test (*p* = 0.0093 for macro-height and *p* = 0.0274 for micro-height). Based on these results, the threshold indices using confluent monolayers were confirmed to define a loss of flatness (Figure 1E). MDCK cell samples with s.d. ≥ 1.0 µm for macro-height, and s.d. ≥ 0.4 µm for micro-height were considered to lose flatness (Figure 1E).

In summary, these data show that confluent MDCK monolayers display a flat, uniform morphology that can be described using s.d. threshold indices. These threshold indices can be used to define a loss of flatness. In the following experiments, we examined the molecular and biophysical processes underlying the maintenance of monolayer flatness.

### Pharmacological inhibition to screen cytoskeleton fibers for potential involvement in macro- or apical micro-height

**FIGURE 2:**
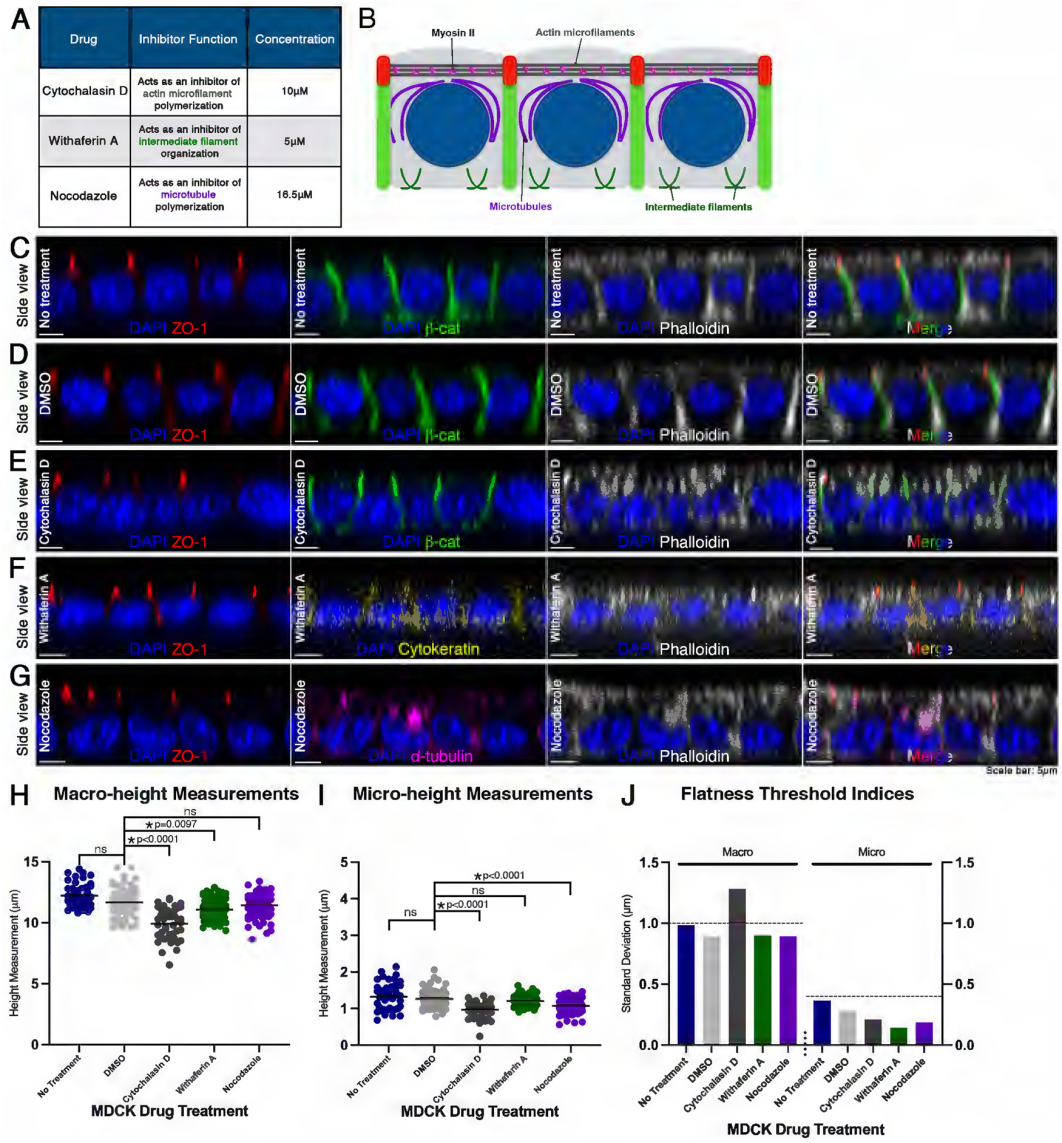
Pharmacological inhibition to screen cytoskeleton fibers for potential involvement in macro- or apical micro-height. (A) Table showing the function and concentration of drugs used to inhibit cytoskeletal polymerization: cytochalasin D, withaferin A, and nocodazole. (B) Schematic of MDCK cells showing localization of actin microfilaments, intermediate filaments, and microtubules. (C) A side view of untreated MDCK cells immunostained with ZO-1 (red), β-catenin (green), and filamentous actin dye, phalloidin (grey), and DNA DAPI (blue) counterstain. (D) As in (C) but with cells treated with DMSO, (E) cytochalasin D. (F) As in (C) but with a withaferin A-treated MDCK cell immunostained with ZO-1 (red), cytokeratin (yellow), phalloidin (grey), and DNA DAPI (blue) counterstain. (G) As in (C), but of nocodazole-treated MDCK cells immunostained with ZO-1 (red), α-tubulin (magenta), phalloidin (grey), and DNA DAPI (blue) counterstain. (H) Measurements of macro-height for individual MDCK cells (*n* = 45 cells for each drug treatment, *n* = 90 cells for withaferin A and nocodazole). Average macro-height for cells with No Treatment, or treated with DMSO, cytochalasin D, withaferin A, and nocodazole were 12.21 µm ± 0.99 µm s.d., 12.20 µm ± 0.89 µm s.d., 9.93µm ± 1.28 µm s.d., 11.09 µm ± 0.90 µm s.d., and 11.43 µm ± 0.89 µm s.d., respectively. (I) As in (H) but of micro-height. Average micro-height for No Treatment, DMSO, cytochalasin D, withaferin A, and nocodazole was 1.31 µm ± 0.27 µm s.d., 1.32 µm ± 0.37 µm s.d., 0.97µm ± 0.21 µm s.d., 1.20 µm ± 0.14 µm s.d., and 1.07 µm ± 0.19 µm s.d., respectively. (J) Flatness threshold indices of macro- and micro-height of treated and control MDCK confluent monolayers. Note: Only cytochalasin D treatment of the MDCK monolayer resulted in a loss of flatness. (Scale bars: 5µm)

To test whether cytoskeletal elements contribute to the regulation of epithelial monolayer flatness, MDCK monolayers were treated with pharmacological inhibitors targeting actin (cytochalasin D), intermediate filaments (withaferin A), and microtubules (nocodazole; Figure 2, A and B). Treated cells were then fixed and labeled with ZO-1, β-catenin, or cytokeratin, or α-tubulin, and phalloidin to visualize epithelial architecture (Figure 2, C–G; Supplemental Figure S3, A and B). Control cells incubated with DMSO showed indistinguishable patterns of polarity markers from untreated MDCK monolayers (*n* = 45 cells each, *n* = 90 for withaferin A and nocodazole; Figure 2, C and D). This was also consistent with macro- and micro-height mean measurements using the Kruskal-Wallis/Dunn's test, and the s.d. of macro- and micro-height was below the threshold indices (H[5, 325] = 78.01 for macro, 64.30 for micro; Figure 2, H and I).

In cytochalasin D-treated cells, ZO-1 and β-catenin localization remained indistinguishable from DMSO control groups (Figure 2E). However, phalloidin staining revealed disruption of filamentous actin into small aggregates, as compared with the uniform and stereotypical appearance in DMSO controls (Figure 2E) (Schliwa, 1982; Stevenson and Begg, 1994). Cytochalasin D-treated cells also displayed disruption of apical and basal actin, and reduced macro- and micro-height (*p* < 0.0001; Figure 2, E, H, I). A decrease in cell height has also been previously reported (Stevenson and Begg, 1994; Wells *et al.*, 1998; Mortensen and Larsson, 2003). The s.d. of macro-height exceeded the threshold index, indicating that treatment with cytochalasin D results in loss of monolayer flatness (Figure 2J).

To directly assess the integrity of the cytoskeletal networks following pharmacological perturbation, we performed IF staining for cytokeratin and α-tubulin to visualize intermediate filaments and microtubules, respectively (Figure 2, F–J). These data confirm that treatment with withaferin A perturbs intermediate filament organization (Figure 2F), while treatment with nocodazole effectively disrupts the microtubule network (Figure 2G). Disruption of intermediate filaments with withaferin A produced changes in macro-, but not micro-height, with s.d. values remaining below the threshold indices used to define loss of flatness (Figure 2J).

In contrast, inhibition of microtubule polymerization with nocodazole resulted in a significant decrease in apical micro-height (Figure 2I; *p* < 0.0001), while overall macro-height and epithelial flatness were largely preserved (Figure 2, H and J). These results indicate that microtubules contribute selectively to apical, micro-height regulation without substantially affecting overall monolayer flatness.

The data indicate that actin and microtubules contribute to apical micro-height regulation, while actin and intermediate filaments contribute to macro-height regulation. These findings support a model in which actin filaments play a role in regulating overall epithelial flatness. This is consistent with the established roles of actin/myosin and microtubule/dynein systems in controlling cell shape and mechanical organization (Quintin *et al.*, 2008; Pollard and Cooper, 2009; Barlan and Gelfand, 2017; Oelz *et al.*, 2018; Huang *et al.*, 2022).

### Defective flatness by inhibition of myosin II

**FIGURE 3:**
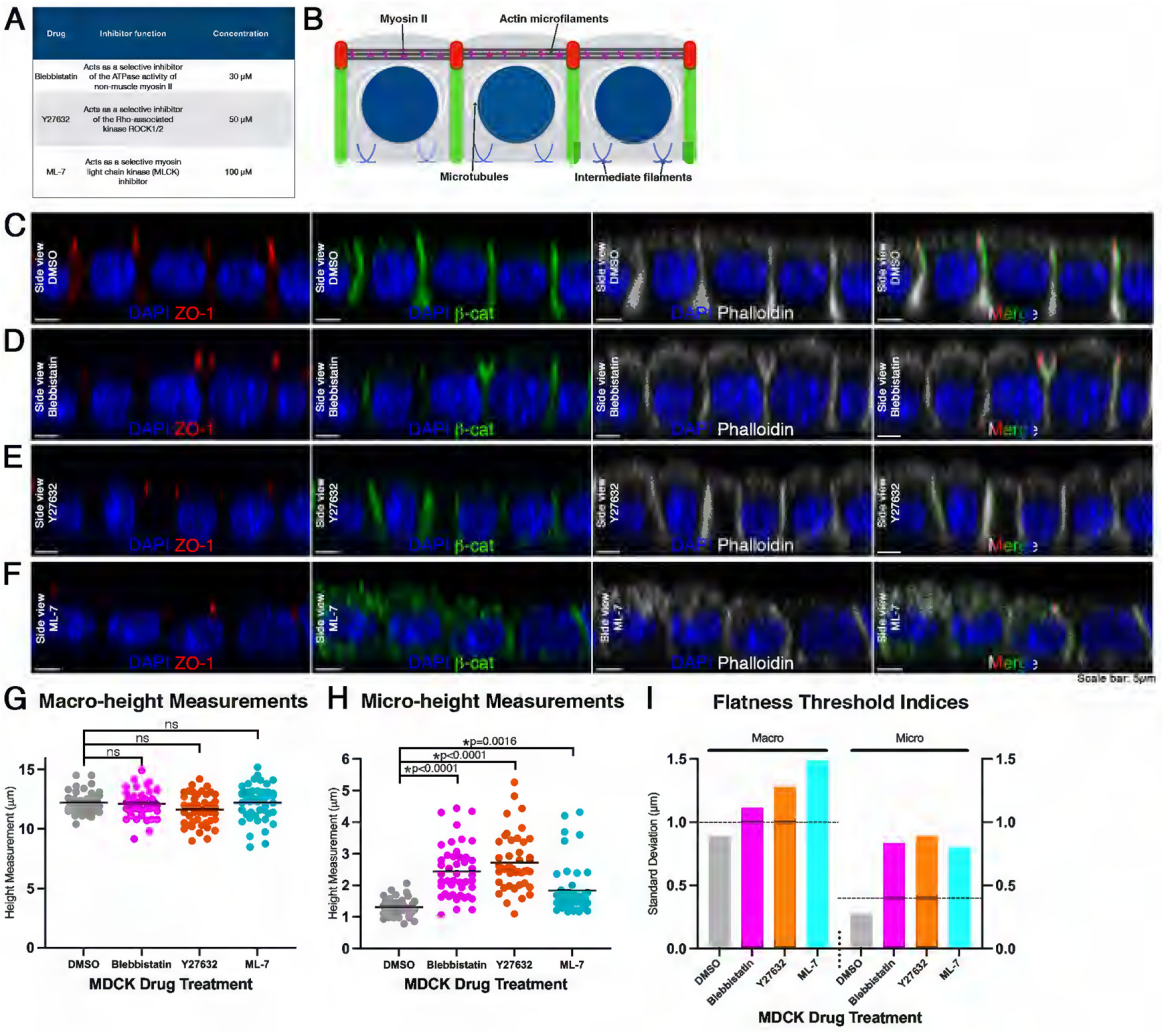
Defective flatness by inhibition of myosin II. (A) Table showing drugs used to target myosin II, inhibitor function, and concentration. (B) Schematic showing the localization of myosin II interaction with actin in an MDCK monolayer. (C) A side view of a single optical section of an MDCK confluent monolayer treated with DMSO, immunostained with ZO-1 (red), β-catenin (green), and filamentous actin dye, phalloidin (grey), and DNA DAPI (blue) counterstain. (D) As in (C) but treated with 30 µM blebbistatin, 50 µM Y27632 (E), and 100 µM ML-7 (F). (G) Individual cell measurements of macro-height for DMSO and drug-treated MDCK monolayers. Average macro-height for DMSO, blebbistatin, Y27632, and ML-7 -treated cells was 12.20 µm ± 0.89 µm s.d., 12.11 µm ± 1.11 µm s.d., 11.61 µm ± 1.28 µm s.d., 12.20 µm ± 1.49 µm s.d., respectively (*n* = 45 cells for each treatment). (H) As in (G) but of micro-height. Average micro-height for DMSO, blebbistatin, Y27632, and ML-7 -treated cells was 1.30 µm ± 0.27 µm s.d., 2.44 µm ± 0.84 µm s.d., 2.72 µm ± 0.89 µm s.d., 1.84 µm ± 0.80 µm s.d., respectively. (I) Flatness threshold indices of macro- and micro-height of treated and control MDCK confluent monolayers. Note: All myosin II inhibitor treatments resulted in a loss of flatness. (Scale bars: 5µm)

We next examined the role of myosin II, the canonical actin partner in the cellular contractile network, in the maintenance of steady-state epithelial flatness. We perturbed the actomyosin contractile network, applying blebbistatin (myosin II ATPase), Y27632 (ROCK), and ML-7 (MLCK) to MDCK monolayers to compromise myosin II motor protein complex function (Kovács *et al.*, 2004; Watanabe *et al.*, 2007; Goeckeler *et al.*, 2008; Cui *et al.*, 2010; Vasquez *et al.*, 2014; Figure 3, A and B).

After treatment with blebbistatin or Y27632, ZO-1 and β-catenin localization patterns remained indistinguishable from DMSO controls (Figure 3, C–E). Treatment with ML-7 resulted in β-catenin localization throughout the cell rather than at cell junctions, differing from DMSO controls (Figure 3F). Individual cells for all three treatments showed an increased apical domain and a dome-like spherical cap of actin as compared with flat apical actin in control DMSO samples (Figure 3, C–F). The average macro-height of cells treated with blebbistatin, Y27632, or ML-7 showed no statistical difference from controls (Figure 3G). However, blebbistatin or Y27632-treated cells exhibited significantly greater average micro-height values compared with DMSO controls (H[4, 180] = 87.19, *p* < 0.0001; Dunn's post-hoc test, *p* < 0.0001 for blebbistatin and Y27632; Figure 3H). ML-7-treated cells showed a slightly milder phenotype, yet average micro-height still differed significantly from DMSO (Dunn's post-hoc test, *p* = 0.0016; Figure 3H). For all three treatments, the flatness threshold indices were exceeded for both macro- and micro-height (Figure 3I). These data show that inhibiting myosin II ATPase with blebbistatin, or ROCK function with Y27632, or MLCK function with ML-7 results in overall loss of MDCK monolayer flatness.

The observation of apical domes with our myosin drug perturbations indicates that some of the underlying actin cytoarchitecture is disorganized. An important component of the apical morphology of MDCK cells is microvilli (Klingner *et al.*, 2014; Gaeta *et al.*, 2021; Sharkova *et al.*, 2023). Microvilli are actin filament-based structures that lack myosin II along their length, with myosin II instead enriched in the underlying filamentous network, or terminal web (Mooseker and Tilney, 1975; Hirokawa *et al*., 1982; Chinowsky *et al.*, 2020). To investigate whether microvilli are disorganized in our MDCK monolayers treated with ML-7, the myosin II treatment that resulted in the mildest phenotype of apical domes, we performed Transmission Electron Microscopy (TEM). TEM analysis revealed a decrease in microvilli number for ML-7-treated MDCK cells as compared with DMSO controls (Supplemental Figure S4, A and B). ML-7-treated cells had an average of 6.75 ± 3.54 s.d. microvilli, while DMSO controls had an average of 35.88 ± 6.13 s.d. microvilli (*n* = 8 cells for each treatment; *t* = 11.64; *p* < 0.0001; Supplemental Figure S4C). As actin-based microvilli decreased in ML-7-treated cells, these results suggest a potential noncontractile role for myosin II, where perturbations of MLCK activity result in loss of flatness via microvilli (Chinowsky *et al.*, 2020; Lombardo *et al.*, 2024).

We recognize that pharmacological treatments may induce off-target effects, and thus the observed outcomes should be interpreted carefully, as they may not be exclusively attributable to the intended actions of the drugs. Therefore, precise genetic perturbations of myosin II are necessary.

### Transgenic perturbation of myosin II alters flatness

To investigate myosin II roles in the actomyosin contractile complex to regulate flatness, we caused an imbalance in contractile dynamics. MLCP has both a regulatory/myosin phosphatase targeting subunit (MYPT1) and a catalytic subunit (PP1δ) (Koga and Ikebe, 2008; Shibata *et al.*, 2015). The *MYPT1^T696A;^ ^T853A^* mutant has two point mutations, T696A and T853A, in the catalytic region of the PP1δ; MLCP is locked in its active conformation (Koga and Ikebe, 2008; Álvarez-Santos *et al.*, 2020). Constitutively active MLCP will cause MLC inactivation and result in an imbalance of relaxation and contraction of myosin (Khromov *et al.*, 2009; Khasnis *et al.*, 2014). We used a transient transfection model for introducing the *MYPT1^T696A;^ ^T853A^* variant into MDCK cells and comparing mutant and wild-type cells.

**FIGURE 4:**
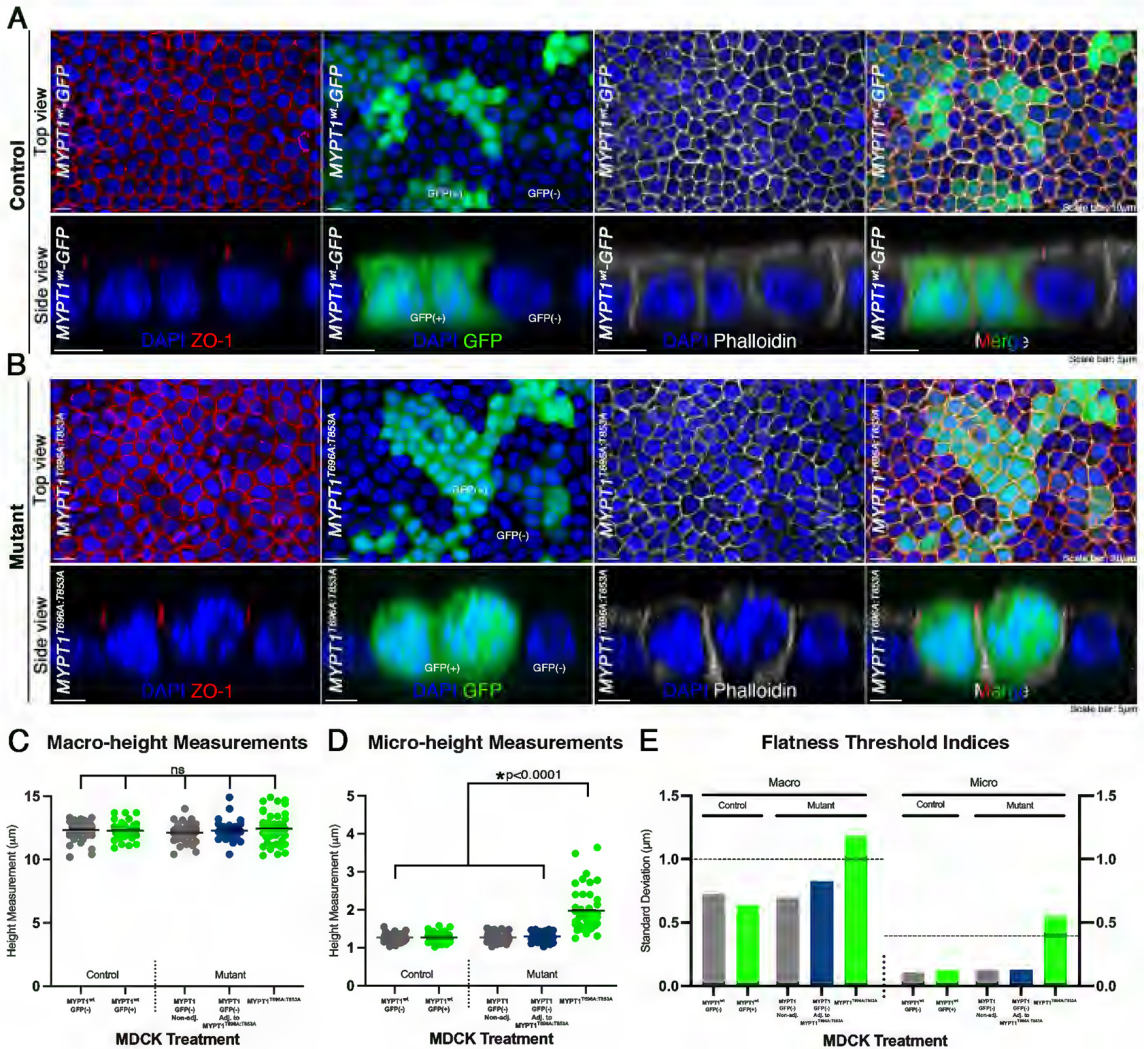
Transgenic perturbation of myosin II alters flatness. (A) Top and side views of stacked confocal optical sections of a control MDCK confluent monolayer transfected with MYPT1^wt^-GFP (green), immunostained with ZO-1 (red), and filamentous actin dye, phalloidin (grey), and DNA DAPI (blue) counterstain. (B) As in (A) but of *MYPT1^T696A;^ ^T853A^* mutant MDCK lines. (C) Individual measurements of macro-height for MYPT1^wt^-GFP control cells (left of vertical dashed line), and *MYPT1^T696A;^ ^T853A^* mutant cells (right of vertical dashed line). Average macro-height measurements for MYPT1^wt^-GFP control cells were 12.30 µm ± 0.77 µm s.d., 12.46 µm ± 0.71 µm s.d., and 12.27 µm ± 0.63 µm s.d. for GFP(−), GFP(−) adjacent to GFP(+), and GFP(+) cells, respectively. Average macro-height for *MYPT1^T696A;T853A^* cells was 12.20 µm ± 0.65 µm s.d., 12.41 µm ± 0.80 µm s.d., and 12.42 µm ± 1.18 µm s.d. for GFP(−), GFP(−) adjacent to *MYPT1^T696A;T853A^* and GFP(+) *MYPT1^T696A;T853A^* respectively. (D) As in (C) but of micro-height for control MYPT1^wt^-GFP cells 1.27 µm ± 0.10 µm s.d., 1.27 µm ± 0.08 µm s.d., and 1.27 µm ± 0.12 µm s.d., respectively. Average micro-height for mutant *MYPT1^T696A;^ ^T853A^* cells was 1.34 µm ± 0.15 µm s.d., 1.38 µm ± 0.14 µm s.d., and 1.97 µm ± 0.55 µm s.d., respectively. A Kruskal-Wallis statistical test was performed to compare mean macro- or micro-height between all groups (H[5, 210] = 93.94, *p* = 0.4555 for macro-height, *p* < 0.0001 for micro-height; Dunn's post-hoc test, *p* < 0.0001 for all treatments compared with *MYPT1^T696A;^ ^T853A^*). Unequal variance was confirmed using Levene's test (*p* = 0.0010 for macro-height, *p* < 0.0001 for micro-height). (E) Flatness threshold indices of macro- and micro-height of treated and control MDCK confluent monolayers. Note: Only mutant *MYPT1^T696A;^ ^T853A^* cells resulted in a loss of flatness. GFP(−) cells adjacent to *MYPT1^T696A;^ ^T853A^* cells remained flat, suggesting that maintaining flatness is a cell-autonomous event. (Scale bars: 10 µm, 20 µm, and 5 µm).

Transiently transfected MYPT1^wt^-GFP MDCK cells exhibited flatness within the monolayer and were used as controls (Figure 4A). Control MYPT1^wt^-GFP cells were categorized as GFP(+) cells that expressed the plasmid and GFP, and GFP(−) cells that did not express the plasmid. Macro- and micro-height measurements showed no significant difference between GFP(−) and GFP(+) cells, and s.d. threshold indices were not exceeded (Figure 4, C–E). These data show that MDCK cells transiently transfected with our control MYPT1^wt^-GFP plasmid retained flatness and served as an appropriate control for comparison of the mutant *MYPT1^T696A;^ ^T853A^* cell line.

We divided the monolayers treated with the mutant plasmid into three groups: (1) GFP(+) *MYPT1^T696A;^ ^T853A^* cells expressing the plasmid with both point mutations, (2) adjacent GFP(−) cells not expressing the plasmid, and (3) non-adjacent GFP(−) cells also lacking plasmid expression. Similar to cells treated with myosin II complex inhibitors (Figure 3), mutant *MYPT1^T696A;^ ^T853A^* cells showed an increased apical domain and dome-like spherical actin caps (Figure 4B), while both adjacent and non-adjacent GFP(−) cells showed a similar flat morphology to our controls (Figure 4, A and B). Average macro-height of *MYPT1^T696A;^ ^T853A^* cells showed no statistical difference from adjacent or non-adjacent GFP(−) cells, nor from GFP(+) and GFP(−) MYPT1^wt^-GFP control cells (Figure 4C). *MYPT1^T696A;T853A^* cells exhibited significantly increased micro-height compared with adjacent and non-adjacent GFP(−) cells, as well as control GFP(+) and GFP(−) cells (H[5, 210] = 93.94, *p* < 0.0001; Dunn's post-hoc test, *p* < 0.0001 for all treatments compared with *MYPT1^T696A;T853A^*; Figure 4D). Loss of flatness and an increased apical domain were observed exclusively in *MYPT1^T696A;^ ^T853A^* cells, whereas all other groups remained flat (Figure 4, A, B, and E). Taken together, the loss of flatness for genetic and drug disruptions of MLCP and MLCK (Figure 3), respectively, provides supportive evidence that myosin II morphodynamics may regulate flatness in a steady-state epithelium.

These data also show that alterations to macro- or micro-height by genetic disruption of MLCP dynamics are limited to the transfected cells and do not significantly influence adjacent cells. Transient transfection creates a mosaic of plasmid-expressing and non-expressing cells within the same monolayer, enabling us to assess whether cellular perturbations act autonomously or influence neighboring cells, non-autonomously (Figure 4; Supplemental Figure S5). GFP(−) cells adjacent to and surrounded by GFP(+) *MYPT1^T696A;^ ^T853A^*-mutant cells retain junctional contacts with multiple neighboring mutant cells (Supplemental Figure S5). In cases where GFP(−) cells were bordered by multiple GFP(+) *MYPT1^T696A;^ ^T853A^*-mutant neighbors, these cells still maintained normal micro-height under these conditions (Supplemental Figure S5), supporting the idea that the MYPT1-dependent micro-height phenotype is primarily cell autonomous. The available data suggest that the increased micro-height phenotype does not propagate to neighboring GFP(−) cells and is primarily cell-autonomous.

### Intracellular Ca^2+^ (iCa^2+^) and maintenance of apical flatness in a steady-state epithelium

Intracellular calcium (iCa^2+^) is an important regulator of many signaling pathways and cellular functions (Bers, 2008; Bagur & Hajnóczky, 2017; Moccia *et al.*, 2023). In particular, iCa^2+^ is a key secondary messenger that helps govern cytoskeletal function, including the actomyosin network, in several tissues and organ systems (Castillo *et al.*, 1998; Yerna *et al.*, 1979; Antunes *et al.*, 2013; Christodoulou and Skourides, 2015; Kuo and Ehrlich, 2015; Sahu *et al.*, 2017; Suzuki *et al.*, 2017). It is unknown whether iCa^2+^ abundance in an epithelial cell contributes to the maintenance of apical flatness.

**FIGURE 5:**
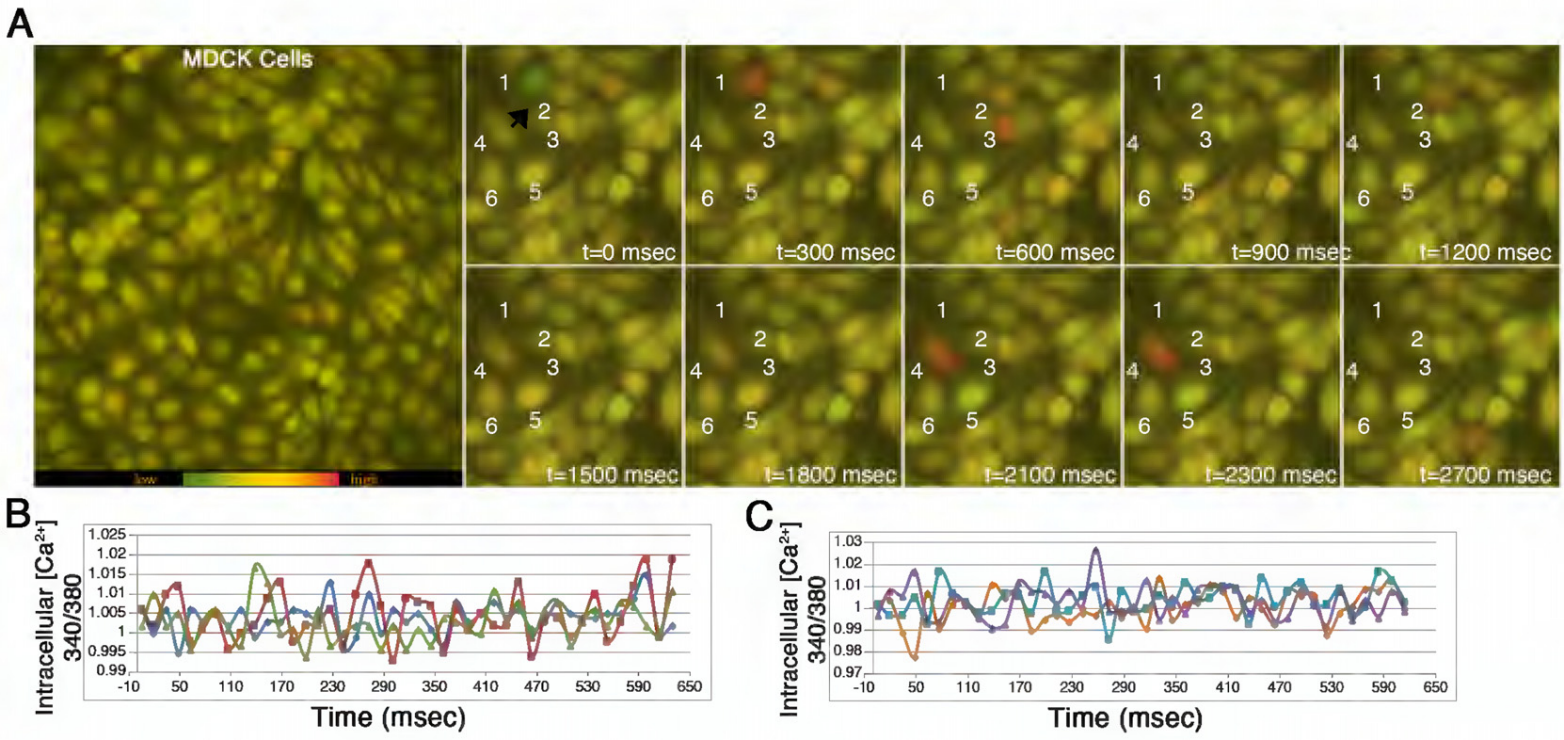
Intracellular Ca^2+^ (iCa^2+^) and maintenance of flatness in a steady-state epithelium. (A) Top view of a time-lapse movie of an MDCK confluent monolayer incubated with 5 µM of Fura-2 dye showing intracellular calcium (iCa^2+^) levels. Selected sequential time frames are shown. (B and C) iCa^2+^ measurements (340 nm/380 nm) of six individual cells (different colored lines).

Therefore, we examined changes in iCa^2+^ in individual cells in our MDCK monolayers. We took advantage of a calcium indicator dye, Fura-2, to perform fluorometric calcium imaging (Barreto-Chang and Dolmetsch, 2009). The fluorescence intensity of the ratio of Fura-2 340/380 nm can be used to calculate iCa^2+^ concentrations (Paredes *et al.*, 2008). Real-time calcium imaging showed rapid, intermittent calcium transients with high [iCa^2+^] appearing as red fluorescence and low [iCa^2+^] as green (Figure 5A; Supplemental Movie S1). With [iCa^2+^] tracked over time, the imaging revealed asynchronous calcium oscillations among adjacent cells in the monolayer (*n* = 6 cells; three samples total; Figure 5, A–C; Supplemental Movie S1). The asynchronous iCa^2+^ oscillations across the monolayer show that individual cells regulate their [iCa^2+^] in an uncoordinated and independent manner from juxtaposed cells. The data support a model of spatio-temporal asynchronous iCa^2+^ oscillations that may play a role in balancing actomyosin dynamics across the monolayer to maintain flatness.

### Association between apical flatness and the ECM

Previous studies have revealed that lateral factors within cells, as well as stiffness of the basal surface, can function to regulate cell height (Cai and Mostov, 2012; Kondo and Hayashi, 2015; Tang, 2017; Janmey *et al.*, 2020; Töpfer, 2023; Perez-Tirado *et al.*, 2025). The ECM regulates many aspects and properties of epithelial sheet morphogenesis, such as apico-basal polarity, cell proliferation, differentiation, and death; serving to establish and maintain proper tissue function (Frantz *et al.*, 2010; Rozario and DeSimone, 2010; Diaz-de-la-Loza *et al.*, 2018; Muncie and Weaver, 2018; Kozyrina *et al.*, 2020; Perez-Tirado *et al.*, 2025). However, it is unknown whether the ECM plays a role in defining the apical surface geometry of steady-state epithelial sheets.

**FIGURE 6:**
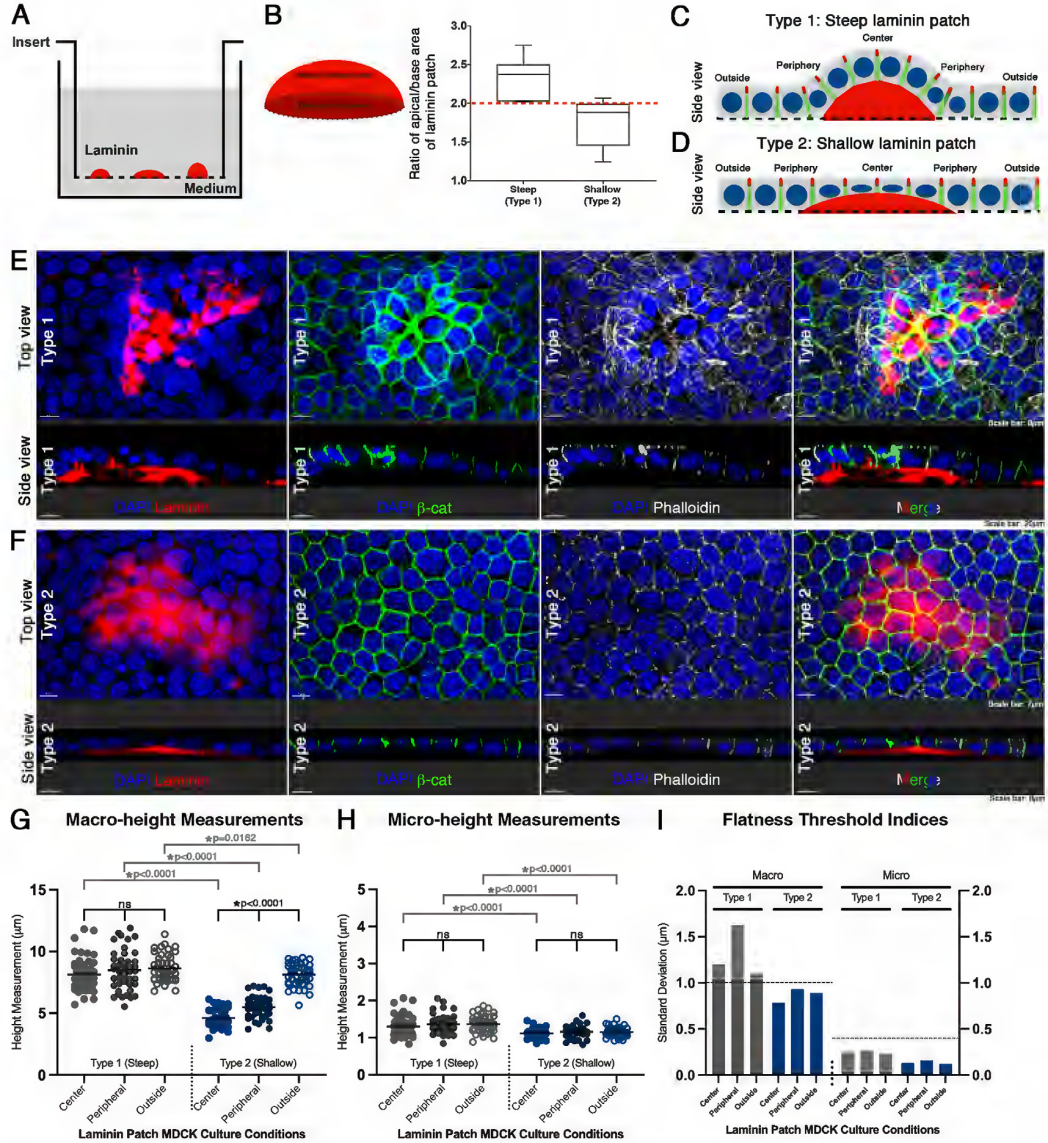
Association between apical flatness and the extracellular matrix (ECM). (A) Schematic of Rhodamine-laminin patches on Transwell membranes before MDCK cell culture. (B) Quantification of steep and shallow Rhodamine-laminin patches. The ratio of apical/basal area of the laminin patch delineated the two categories of steep and shallow patches. “Steep” patches had a ratio of 2.35 ± 0.26 s.d., and “shallow” patches had a ratio of 1.74 ± 0.31 s.d. Note: Both steep/shallow patches were clearly delineated by a ratio that was either > or < 2, with the ‘steep’ patch ratio > 2, and “shallow” patch ratio < 2 (*n* = 16 laminin patches). (C, C’) Two types of epithelial response in morphology are based on the steepness of the laminin patch. Cells with complete, partial, or little to no basement membrane positioned on the patch were defined as center, peripheral, or outside, respectively. (D) Top and side views of stacked confocal optical sections of a Type 1 MDCK confluent monolayer immunostained with β-catenin (green), filamentous actin dye, phallodin (grey), and a DNA DAPI (blue) counterstain when grown on a “steep” Rhodamine-laminin patch (red). (E) As in (D) but of a Type 2 MDCK confluent monolayer growing on a “shallow” Rhodamine-laminin patch (red). (F) Macro-height measurements for Type 1 (grey) and Type 2 (blue) monolayers. Individual cell height analysis was performed based on the relative position on the laminin patch. Average macro-height of center, peripheral, or outside cells was 8.10 µm, 8.42 µm, and 8.64, respectively (*n* = 149 cells). For Type 2 cells, the average macro-height for center, peripheral, and outside cells was 4.60 µm s.d., 5.47 µm, and 8.12 µm, respectively (*n* = 117 cells). (G) As in (F), but of micro-height measurements. For Type 1 cells, average micro-height measurements for center, peripheral, and outside cells were 1.29 µm, 1.36 µm, and 1.37 µm, respectively (*n* = 149 cells). For Type 2 cells, the average micro-height for the center, peripheral, and outside cells was 1.12 µm, 1.16 µm, and 1.15 µm, respectively (*n* = 117 cells). (H) Standard deviations of macro- and micro-height of Types 1 (steep) and 2 (shallow) MDCK confluent monolayers. Flatness threshold indices established in Figure 1 are indicated with horizontal dashed lines. Note: Type 1 epithelium loses flatness, while Type 2 epithelium maintains flatness by modulating individual cellular behaviors on the laminin patch. (Scale bars: 8 µm, 20 µm, 7 µm).

To test this possibility, we engineered a basement membrane with a patched deposition of fluorescently labeled laminin on transwell filters to represent an uneven ECM (Figure 6A). Under our culture conditions, the patched laminin still allowed MDCK cells to successfully establish a confluent monolayer (*n* = 6 samples). Laminin patches were classified as “steep” or “shallow” based on the ratio of apical to basal surface area. Steep laminin patches had a ratio of 2.35 ± 0.26 s.d. for apical/basal surface area. Shallow laminin patches had a ratio of 1.74 ± 0.31 s.d. for apical/basal surface area (Figure 6B). Both types of epithelium were clearly delineated by a laminin patch ratio that was either larger (steep) or smaller (shallow) than 2 (*n* = 16 laminin patches; Figure 6B).

Epithelia were classified into Type 1 or 2. Type 1 epithelium was associated with steep laminin patches, while Type 2 was associated with shallow laminin patches (Figure 6, B–D). Type 1 epithelium displayed a wavy surface tissue morphology that traveled along the contours of the steep laminin patch (*n* = 3 samples; Figure 6, C and E). Type 2 epithelium had a flat tissue geometry that modulated to adapt along the shallow laminin patches (*n* = 3 samples; Figure 6, D and F).

To determine the flatness of the two types of epithelia, individual cell height analysis was performed based on the relative position on the laminin patch. Cells with complete, partial, or little to no basement membrane positioned on the patch were defined as center, peripheral, or outside, respectively (Figure 6, C and D). We quantified macro- and micro-height using β-catenin staining (Supplemental Figure S2).

Macro-height measurements of Type 1 (steep) epithelial cells of the center, peripheral, or outside were 8.10 µm, 8.42 µm, and 8.64 µm, respectively (*n* = 149 cells; Figure 6G). A Kruskal-Wallis statistical test was performed to compare mean macro-height measurements between groups of Type 1 cells (H(2, 153) = 5.097, *p* = 0.0782), with unequal variance confirmed using Levene's statistical test (*p* = 0.0177). Average micro-height measurements for center, peripheral, and outside cells were 1.29 µm, 1.36 µm, and 1.37 µm, respectively (Figure 6H). A one-way ANOVA statistical test was performed to compare mean micro-height measurements between groups of Type 1 cells (F [2, 146] = 1.537, *p* = 0.2185), with equal variance confirmed using Levene's statistical test (*p* = 0.8414). Average macro- and micro-height measurements were not statistically different between center, peripheral, and outside cells of Type 1 epithelium (Figure 6, G and H).

Although average macro-heights for Type 1 cells were similar (Figure 6G), the s.d. values are above the threshold index of flatness of 1.0µm, indicating that flatness was lost (Figure 6I). These data demonstrate that Type 1 epithelium loses its flatness by developing increased cell height variability among central, peripheral, and outside cells.

For Type 2 (shallow) epithelium, average macro-height for center, peripheral, and outside cells was 4.60 µm, 5.47 µm, and 8.12 µm, respectively (*n* = 117 cells; Figure 6G). The data show that macro-height is significantly different between center, peripheral, and outside cells in Type 2 epithelia (One-way ANOVA, F[2, 114] = 177.5, *p* < 0.0001; Levene's test, *p* = 0.7065). However, the average micro-height measurements were not significantly different for the center, peripheral, and outside cells were 1.12 µm, 1.16 µm, and 1.15 µm, respectively (One-way ANOVA, F[2, 114] = 0.9336, *p* = 0.3961; Levene's test, *p* = 0.5907; Figure 6H). The s.d. of macro- and micro-height of Type 2 cells did not exceed the flatness threshold indices, indicating that the monolayer remained flat (Figure 6I). These results reveal a striking pattern: Type 2 epithelia on a shallow laminin patch adapt to maintain a uniform tissue flatness by decreasing their individual cell height, or macro-height.

In summary, these data show that flatness is lost in Type 1 epithelium on a steep laminin patch, and is maintained for Type 2 epithelium on a shallow laminin patch. Measurements of macro- and micro-height for Type 2 center, peripheral, and outside cells were significantly shorter than Type 1 counterparts confirming the two distinct monolayer behaviors defined by steep/shallow laminin patches (six comparisons, four Mann-Whitney U tests (U = 563, 378, 407.5, and 60, all *p* < 0.0001) and two Student's t-tests (*t* = 15.54, *p* < 0.0001; *t* = 2.452, *p* = 0.0162; Figure 6, G and H). The apical surface of the two different types of epithelial sheet was continuous regardless of basement membrane evenness. These observations suggest that it is not the ECM component, but rather the steepness and topography of the ECM that determines whether the steady-state epithelial sheet maintains a constant height along the patch, or decreases to adapt to laminin topography. These data support that apical surface flatness is differentially regulated by the basement membrane after a steepness threshold. Our findings suggest that individual MDCK cells can detect substrate steepness and respond by regulating their height at both macro- and microlevels.

## DISCUSSION

In this study, we employed several pharmacological agents to disrupt actin and myosin networks in a steady-state epithelium with the goal of understanding what forces are acting to maintain the flatness of epithelial sheets. Although there are individual caveats with each of the methods we used, when considered together, the results hint at some interesting dynamics at play in maintaining a steady-state epithelium.

MDCK cells are derived from canine kidney distal tubule epithelium, which in vivo exhibits a predominantly cuboidal morphology (Cho *et al.*, 1989). The cultured renal epithelial cell heights we measure under confluent conditions are consistent with a cuboidal organization rather than a squamous phenotype (Cai and Mostov, 2012). In contrast, subconfluent MDCK cells display increased variability in height and surface contour, which may resemble a less organized or partially spread epithelial state rather than a physiologically defined squamous epithelium (Dukes *et al.*, 2011; Chmiel and Gardel, 2022).

To quantify epithelial surface organization, we established a definition of flatness using confluent MDCK monolayers. Flatness was evaluated at both tissue, macro-, and cellular, microlevels based on height variability across the epithelial surface. Confluent MDCK cells exhibited consistent uniform junctional organization marked by ZO-1, β-catenin, and phalloidin labeling. Using these measurements, we defined a flatness index based on the SD of tissue and cellular height variation. Subconfluent MDCK cells, lacking full junctional organization, showed significantly higher variability, validating this metric as a measure of apical irregularity. Monolayers with s.d. ≥1.0 µm (macro), or ≥0.4 µm (micro), were thus considered to have lost flatness. This framework provided a quantitative standard or threshold for assessing epithelial morphology and identifying disruptions in flatness following drug and genetic perturbations.

Epithelia in vivo exhibit a wide range of morphology, including squamous (such as endothelium), cuboidal (such as renal tubules), and columnar (such as intestinal epithelium). Each of these epithelia is associated with distinct mechanical and functional environments and processes (Bartle *et al.*, 2018). We do not necessarily expect absolute cell height or flatness indices to be identical across these epithelial geometries. Rather, we propose that our flatness indices are best interpreted as measures of monolayer uniformity rather than absolute cell height. Flatness reflects how consistently cells maintain a shared apical surface within a given epithelial environment, independent of whether the tissue is squamous, cuboidal, or columnar. The flatness index may be a geometry-independent metric of epithelial surface uniformity. Future studies comparing epithelial types with defined in vivo geometries would further test how broadly this metric applies across tissues.

Our inhibitor experiments were designed to interrogate cytoskeletal fibers specifically within an established MDCK epithelial monolayer. Interestingly, we did not observe any scenario in which only micro-height s.d. exceeded the flatness threshold while macro-height s.d. remained below it, indicating that microscale fluctuations alone do not drive loss of monolayer flatness. Instead, our cytoskeletal perturbation experiments showed that cytochalasin D, which disrupts actin, altered both macro- and micro-heights and led to loss of flatness. Withaferin A, which disrupts intermediate filaments, showed a decrease in macro-height, whereas nocodazole reduced micro-height without compromising overall flatness (Figure 2).

To further investigate underlying cellular processes that may contribute to these responses, we pharmacologically inhibited actin and several myosin II-associated enzymes. These perturbations resulted in defective flatness, including increased micro-height and spherical “domes” of actin at the apical surface (Figures 2 and 3). This phenotype was preserved following higher-specificity genetic perturbation with a *MYPT1^T696A;^ ^T853A^* mutant cell line, containing point mutations T696A and T853A to the MYPT1 regulatory subunit of MLCP. Interestingly, the transient transfection experiments also revealed that these mutations acted cell-autonomously and exhibited no distinguishable effects on neighboring cells. Micro-height regulation is largely cell autonomous under the conditions tested, while we acknowledge that a basal level of epithelial connectivity may still contribute to overall tissue mechanics. Together, our data support a model in which MYPT1-dependent regulation of apical micro-height operates in a predominantly cell-autonomous manner, even within a mechanically integrated epithelial sheet.

Live imaging of iCa^2+^ fluorescence showed transient, asynchronous iCa^2+^ oscillations across the monolayer, suggesting a potential role of calcium, and further demonstrating cell autonomy in the process of maintaining epithelial flatness.

These data reveal several potential roles of myosin II in the maintenance of steady-state epithelial flatness. Although myosin II has been known for its roles in the actomyosin contractile complex, it may also have non-contractile functions that are involved in flatness regulation of an epithelium. One potential role of myosin II in apical flatness is its influence on microvilli at the surface of MDCK cells. Pharmacological inhibition targeting myosin II ATPase, ROCK, and MLCK resulted in defective flatness, including increased micro-height and spherical “domes” of actin (Figure 3). Apical surface area enlargement and a dome-like spherical cap can be generated through microvilli retraction and buildup of apical actin (Ivanov *et al.*, 2005; Jerdeva *et al.*, 2005; Pietuch et al, 2013; Odenwald *et al.*, 2018; Chinowski *et al.*, 2020). Using TEM, we found a decrease in the number of microvilli in MDCK cells treated with ML-7 (Supplemental Figure S4), although the sample size analyzed is small. Future studies would benefit from increasing the sample size and examining the effects of blebbistatin or Y27632 treatment on microvilli number.

Our *MYPT1^T696A;^ ^T853A^* mutant cells displayed increased micro-height and spherical caps of actin (Figure 4), similar to the results of our myosin II inhibitor treatments. These data taken together support the model that myosin II may be functioning in multiple facets of flatness regulation at the apical surface of epithelial cells: as a component in the actomyosin-contractile complex, and in having a non-contractile role in microvilli architecture. The point mutations T696A and T853A are known to reduce phosphorylation of MYPT1 by ROCK, thus contributing to reduced MLCP inhibition and myosin II activity (Koga and Ikebe, 2008; Khromov *et al.*, 2009; Khasnis *et al.*, 2014; Álvarez-Santos *et al.*, 2020). Importantly, no significant differences were observed in macro- or micro-height in GFP(−) cells either directly adjacent to, or entirely bordered by, *MYPT1^T696A;^ ^T853A^* mutant cells, suggesting that maintenance of flatness is a cell-autonomous process (Figure 4, Supplemental Figure S5). This finding is unexpected, as apical organization is often presumed to be tissue-coordinated, and it provides a mechanism by which epithelial integrity is preserved even when neighboring cells are perturbed.

The conclusions of the study, that actomyosin contractility regulates micro- and macro-height, are supported independently of the iCa^2+^ data. The iCa^2+^ measurements are included to highlight an additional layer of dynamic intracellular signaling that may be compatible with the observed cell-autonomous regulation of apical height. With respect to whether iCa^2+^ fluctuations imply corresponding myosin fluctuations, we acknowledge that our current temporal and spatial resolution does not allow direct coupling of individual iCa^2+^ transients to myosin activity. Demonstrating such causality would require simultaneous high-resolution biosensors for calcium and myosin activity.

Lastly, epithelial height is regulated by both intracellular forces and ECM mechanics (Cai and Mostov, 2012; Neelam *et al.*, 2016; Janmey *et al.*, 2020). To examine whether the ECM topography influences apical geometry, MDCK cells were grown on patched laminin substrates that introduced uneven basement membrane contours. Two epithelial morphologies emerged: Type 1 epithelium on “steep” laminin patches (ratio > 2) formed wavy monolayers contiguous with the laminin contour, while Type 2 epithelium on “shallow” patches (ratio < 2) modulated individual cell heights relative to the laminin patch to maintain flatness. Type 1 epithelia exceeded the macro-height threshold, indicating a loss of apical flatness, whereas Type 2 epithelia remained within the defined standard. Micro-height was similar across all conditions, suggesting local height regulation despite topographical variation. These results demonstrate that ECM steepness, rather than composition, may dictate epithelial height organization: epithelial cells may sense the substrate slope and modulate their height to preserve the overall epithelial architecture of the monolayer. Our finding of previously unknown epithelial tissue behavior in response to substrate steepness may provide new insights into epithelial cell morphology and plasticity and may broaden our understanding of cellular adaptation to complex microenvironments.

This study sheds light on the dynamics of the MLCK and actomyosin complex in the physiological context of a steady-state epithelium. It is significant to understand regulators of apical epithelial cell morphology because loss of flatness has been correlated with tumor metastasis (Coradini *et al.*, 2011; Eisenhoffer and Rosenblatt, 2012; Macara and McCaffrey, 2013; Hannezo *et al.*, 2014; Rodriguez-Boulan and Macara, 2014; Jung *et al.*, 2019; Janiszewska *et al.*, 2020; Ribatti *et al.*, 2020; Peglion and Etienne-Manneville, 2024). By uncovering key mechanisms that govern apical morphology, this work contributes to our understanding of how disruptions in actomyosin dynamics and microvilli organization can drive changes in epithelial cytoarchitecture and function. Understanding how flatness is dynamically regulated may provide insight into epithelial resilience and failure during development, injury, and disease.

## MATERIALS AND METHODS

### Cell preparation and culture

Madin Darby Canine Kidney (MDCK) cells (gift from Keith Mostov, UCSF) were cultured in DMEM complete media (Life Technologies) supplemented with 5% FBS (Life Technologies), 1% penicillin-streptomycin in 10 cm^2^ cell culture plates (Corning) and/or transwell cell culture inserts (Corning).

### Transient transfection of mutant MDCK cell lines

*MYPT1^T696A;^ ^T853A^* mutant MDCK lines were generated by plasmid transfection using the Neon electroporation system (Invitrogen). The insert was cloned using Gibson assembly into the pK.myc-MYPT1 plasmid (#24101, Addgene). Mutations were introduced in the plasmid using the In-Fusion Cloning kit (TakaraBio) primer design approach. Primer pairs covering the cDNA sequences were designed: 5′ GTC TAG AAG ATC AGC ACA GGG AGT AAC ATT GAC T 3′, and 5′ AGT CAA TGT TAC TCC CTG TGC TGA TCT TCT AGA C 3′ for *MYPT1 T695A point mutation*; 5′ GAG AAA AGG AGA TCC GCA GGA GTT TCA TTT TGG ACA CA 3′, and 5′ TGT GTC CAA AAT GAA ACT CCT GCG GAT CTC CTT TTC TC 3′ for *MYPT1 T852A point mutation*. The constructed vectors were then sequenced (Quintara Biosciences) and used for transfection.

### Transepithelial electrical resistance (TEER)

MDCKs were harvested from 10 cm^2^ cell culture plates at a confluency of 70%–80%, and were seeded at a density of 1–5 × 10^5^ cells per insert in the Transwell membrane apparatuses (Corning) to the desired confluence. TEER measurements were taken 24 h after seeding, at each 1 h time point, or 20 h after seeding, at each 4 h time point with the Epithelial Volt/Ohm meter (EVOM) (World Precision Instruments) or ScopeMeter 190 Series (Fluke) in a Biosafety cabinet at room temperature (RT; Supplemental Figure S1, A and B). The background resistance was taken from an insert with media only, subtracted for each TEER reading, and multiplied by the surface area of the filter (1.12 cm^2^).

### Drug treatments

MDCK cells were treated with 10µM of cytochalasin D (C8273, Sigma), or 5µM of withaferin A (W4394, Sigma), or 16.5µM of nocodazole (M1404, Sigma), 30µM blebbistatin (B0560, Sigma) or 50µM Y27632 (sc-281642; Santa Cruz Biotechnology), 100µM ML-7 (ab120848; Abcam), dissolved in DMSO for 2 h in an incubator at 37°C, 5% CO_2_ before processing. Experiments were completed on three individual MDCK monolayer samples for each drug treatment, with the exception of withaferin A and nocodazole treatments having six monolayer samples. In total, 15 cells were measured from each monolayer sample.

### Immunofluorescence

Transwell membranes were transferred to a 12-well plate containing cold phosphate-buffered saline (PBS). The permeable Transwell membranes were then fixed with 4% paraformaldehyde (PFA) for 30–60 min on ice, rinsed 3X with cold PBS, and incubated with permeabilization solution (PFS; 0.7% fish skin gelatin, 0.025% saponin dissolved in PBS) for 1 h at RT.

Membranes were then cut from the original Transwell apparatus using a scalpel. Membranes were then incubated with antibodies to mouse ZO-1 (1:50; Abcam), rabbit β-catenin (1:1000; ab6302, Abcam), stained with Alexa Fluor 647 phalloidin (1:500; A22287, Invitrogen), and DAPI (1:1000; Invitrogen). Secondary antibodies using rabbit Alexa Fluor 488 (1:100; Invitrogen), Alexa Fluor 594 (Invitrogen) were then applied. Following antibody staining, Transwell membranes were then mounted on glass slides (FisherScientific) using Prolong Gold Antifade (Invitrogen), and coverslipped (FisherScientific). Rhodamine-laminin (#LMN01-A, Cytoskeleton) was applied to Transwell membranes. Following antibody staining, Transwell membranes were then mounted on glass slides (FisherScientific) using Prolong Gold Antifade (Invitrogen), and coverslipped (FisherScientific).

### Image capture and acquisition

Fixed MDCK cells were imaged using a confocal microscope (TCS SP2, DM2500; Leica) using a 63X oil immersion objective as previously described (Hua and Mikawa, 2018). Images were reconstructed from confocal optical sections using Imaris software (Bitplane). Brightness and color were adjusted using Photoshop (Adobe Systems).

### Transmission electron microscopy (TEM)

MDCK cells were seeded in Transwell filters, and the Transwell filters were cut into small pieces and put into embedding plastic molds. After overnight adherence, the cells were either treated with DMSO (control) or 100 µM ML-7. The morphology of the MDCK cells was visualized with TEM as described in Heckman *et al.*, (2007).

### Quantification of cell and apical height analysis

MDCK monolayers were reconstructed from confocal optical sections and analyzed using Imaris software (Bitplane). Measurements were conducted using the Imaris measurement tool function on an orthogonal plane from the 3D reconstructed optical sections (Supplemental Figure S2B). For immunofluorescence analysis, fluorescent images were thresholded by highlighting the brightest 80% of the images for all channels. Phalloidin staining was used to determine the maximum height of the cell's apex (Supplemental Figure S2B). The bottom of the cell was defined by Phalloidin exclusion staining along the apical-basal axis, allowing consistent and reproducible height measurements across conditions. Micro-height was measured as the vertical distance from the tricellular junction midpoint to the apex of phalloidin staining (Supplemental Figure S2B). Macro-height was measured as the vertical distance from the midpoint to the cell base (Supplemental Figure S2C).

Plots were generated in Graphpad Prism 10 to present height measurements, treated vs untreated cells, GFP(−), GFP(+) cells for the various experiments, and the ratio of apical/basal area of laminin patches in MDCK culture conditions. Quantification of ECM bumps was completed using Imaris (Bitplane).

### Statistical analysis

Confluent and subconfluent measurements (Figure 1) and cell height measurements grown on laminin patches (Figure 2) were statistically compared using either a parametric or non-parametric test, contingent upon Levene's test for unequal variance between groups. Equal variance prompts a parametric Student's *t* test, while unequal variance prompts a nonparametric Mann-Whitney U-test. Drug-treated samples (Untreated, cytochalasin D, withaferin A, nocodazole; Figure 2), (blebbistatin, Y27632, ML-7; Figure 3), and transfected samples (*MYPT1^T696A;^ ^T853A^*; Figure 4) were statistically compared using either a One-way ANOVA or Kruskal-Wallis test, contingent upon Levene's test for unequal variance between groups. Equal variance prompts a parametric One-Way ANOVA, while unequal variance prompts a non-parametric Kruskal-Wallis test. On Figures 3 and 4, post-hoc tests (Dunnett's test if a one-way ANOVA or Dunn's test if a Kruskal-Wallis test) were performed, allowing multiple comparisons of the treated groups to the DMSO control group.

### Calcium imaging

MDCK cells were grown on 3.5 cm glass dishes (Ibidi) and incubated with 2 µM Fura-2 AM in DMSO at 37°C for 30 min. Cells were then washed with 1X Hank's Balanced Salt Solution (HHBS). Individual cells were excited at 340 nm and 380 nm with emission at 510 nm as a ratiometric indicator for intracellular Ca^2+^concentration measurements. Ex/Em: 340/510 nm can be used to measure Ca^2+^-bound Fura-2, and Ex/Em: 380/510 nm can be used to measure Ca^2+^-free Fura-2, indicating high and low concentrations of Ca^2+^ (Ion Biosciences). Imaging was done at 300 ms intervals with no delay for 20 min. Imaging was captured using an inverted Nikon Ti CSU-X1 Spinning Disk confocal microscope supported by a Photometrics Prime 95B sCMOS camera, and an OkoLab cage incubator with CO_2_ and humidity control with a Plan Apo 40X Objective using Nikon Elements Advance Research software.

## Supporting information





Supporting Video 1 Movie S1 **Live cell imaging of intracellular Ca^2+^ (iCa^2+^) and maintenance of apical flatness in steady state epithelium.** Live imaging video of an MDCK monolayer acquired over 30 minutes at 300 ms intervals stained with Fura-2 calcium dye. High and low [iCa^2+^] are designated in red and green, respectively. Note: iCa^2+^ concentrations oscillate asynchronously among individual MDCK cells across the monolayer. (Scale bars: 2 µm).

## Abbreviations used:

DAPI (4′,6-diamidino-2-phenylindole): fluorescent DNA-binding stain
DMSO: dimethyl sulfoxide
ECM: extracellular matrix
iCa²⁺: intracellular calcium
MDCK: Madin-Darby canine kidney (cells)
MLCK: myosin light chain kinase
MLCP: myosin light chain phosphatase
MYPT1: myosin phosphatase target subunit 1
TEER: transepithelial electrical resistance
TEM: transmission electron microscopy
ZO-1: zonula occludens-1.
