# Supplementary material for: Steady-state epithelial apical flatness is characterized by MLCK morphodynamics and asynchronous Ca2+ oscillations, but not by underlying ECM geometry
Source: Mol Biol Cell. 2026 Apr 14;37(5):ar45. doi: 10.1091/mbc.E25-12-0583 (PMC13244296; doi:10.1091/mbc.E25-12-0583)
Supplement: Supplementary file 1 [file mbc-37-ar45-s001.pdf]

Supplemental Materials

Molecular Biology of the Cell

Wu *et al*.

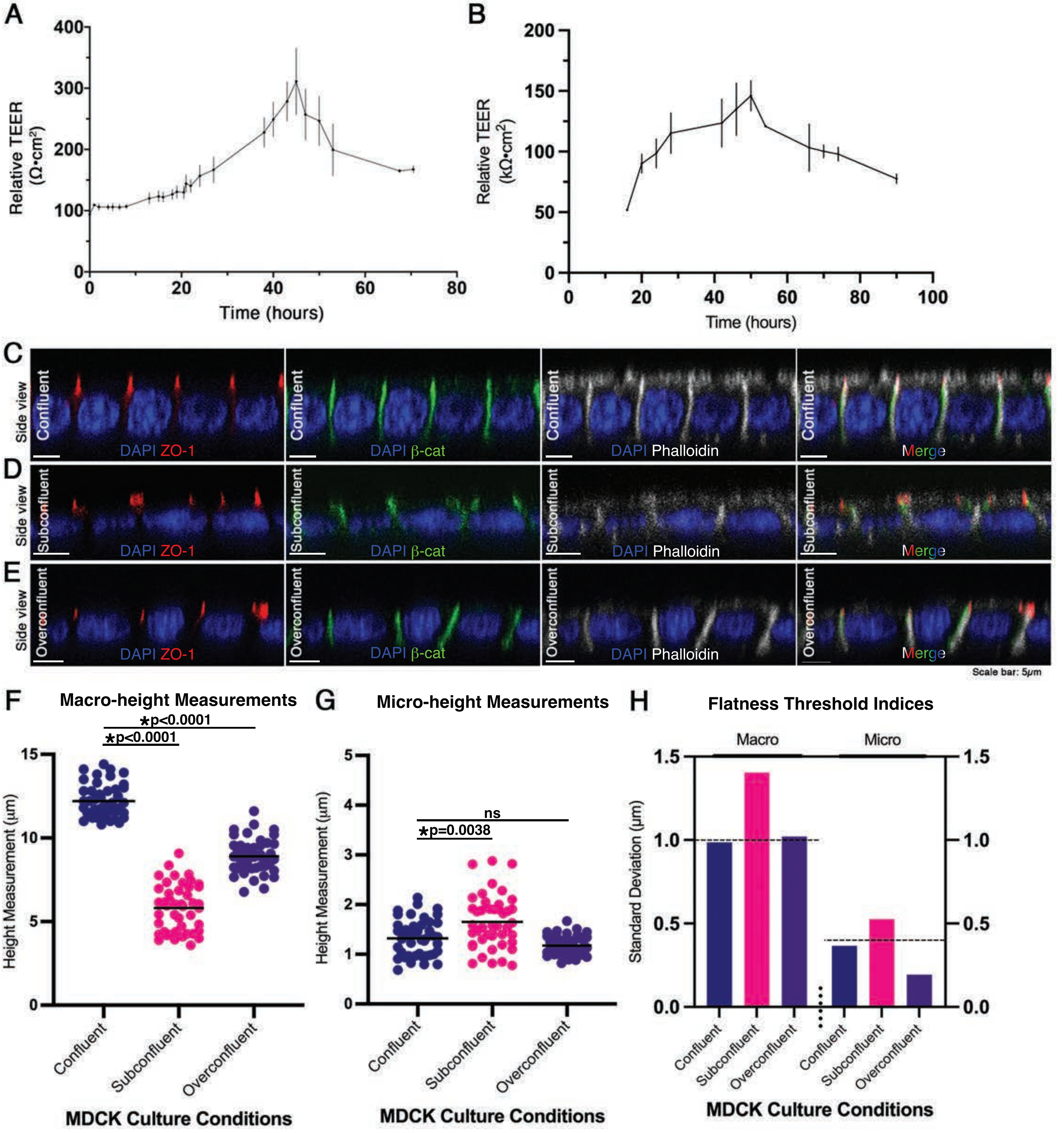

Wu, Herrmann, Hyer, Hua, and Mikawa sFigure 1

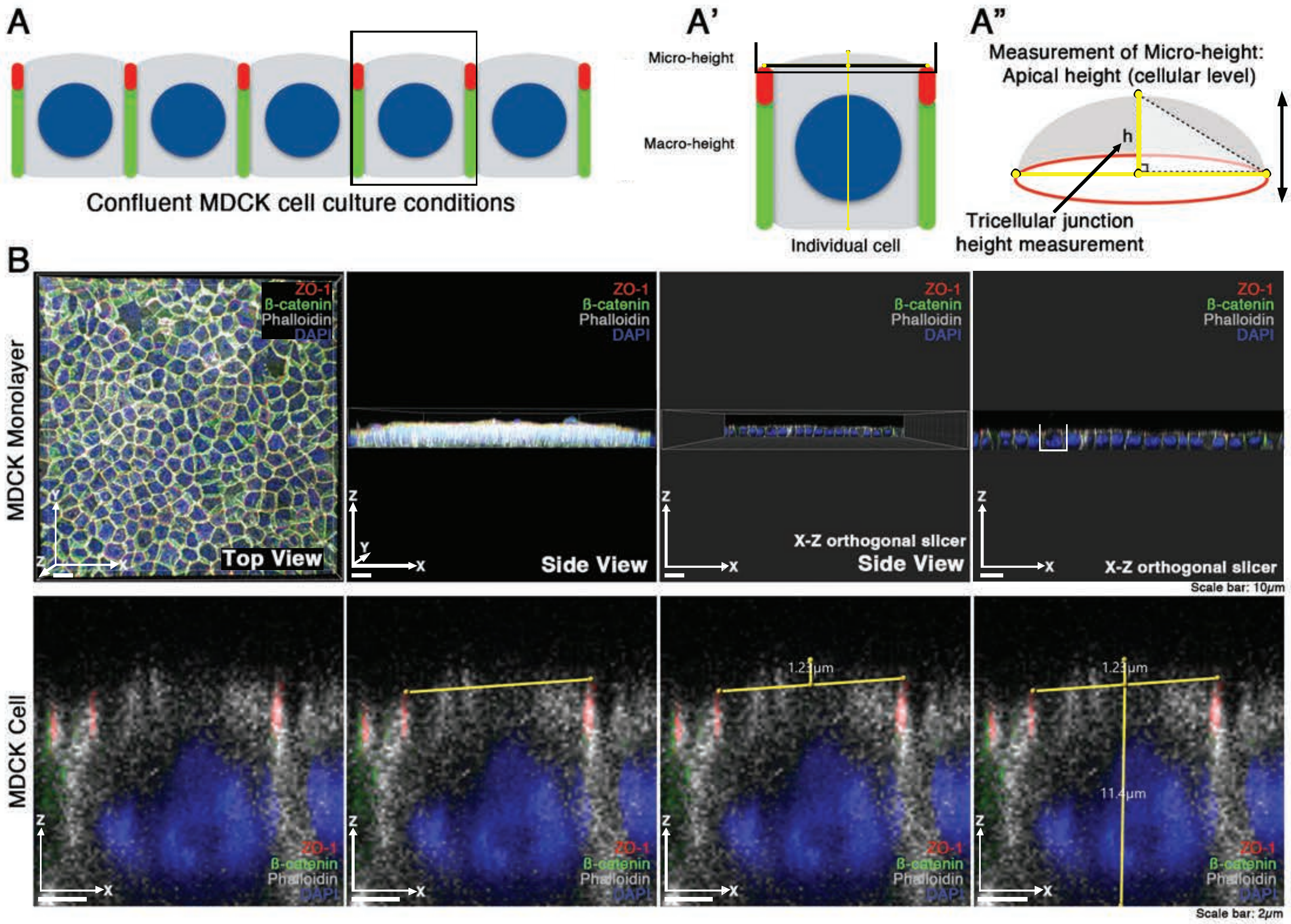

Wu, Herrmann, Hyer, Hua, and Mikawa sFigure 2

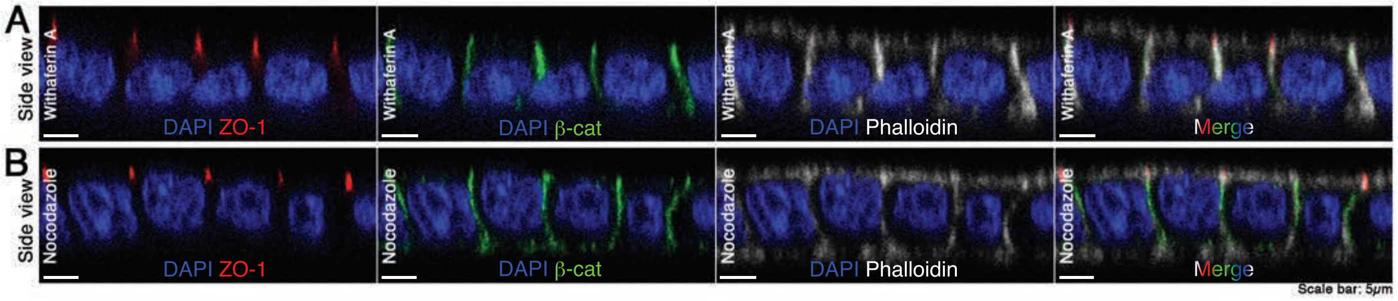

Wu, Herrmann, Hyer, Hua, and Mikawa sFigure 3

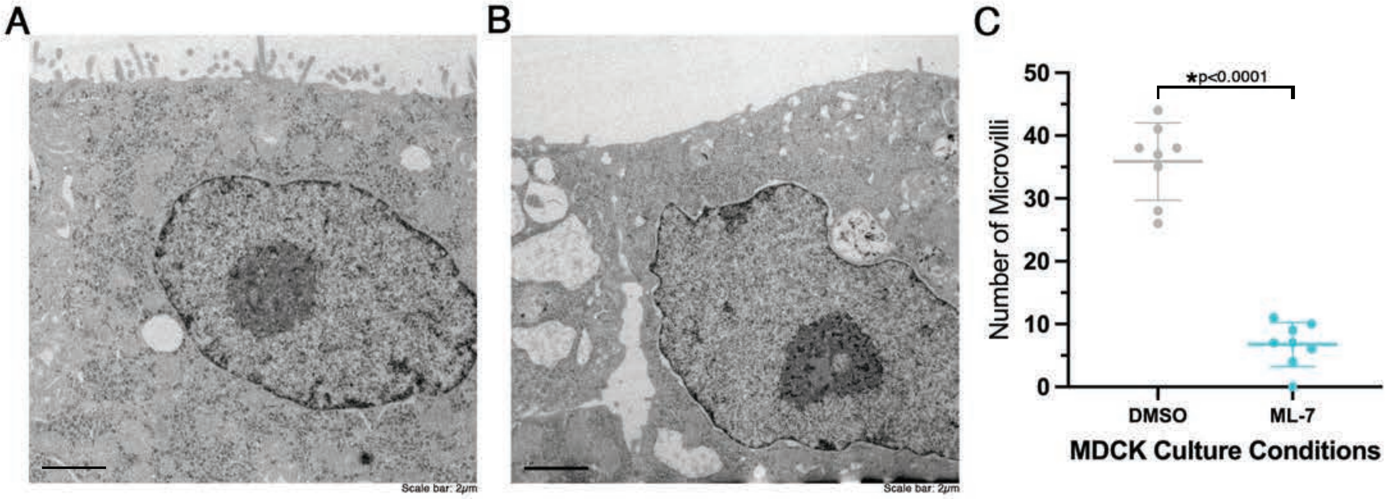

Wu, Herrmann, Hyer, Hua, and Mikawa sFigure 4

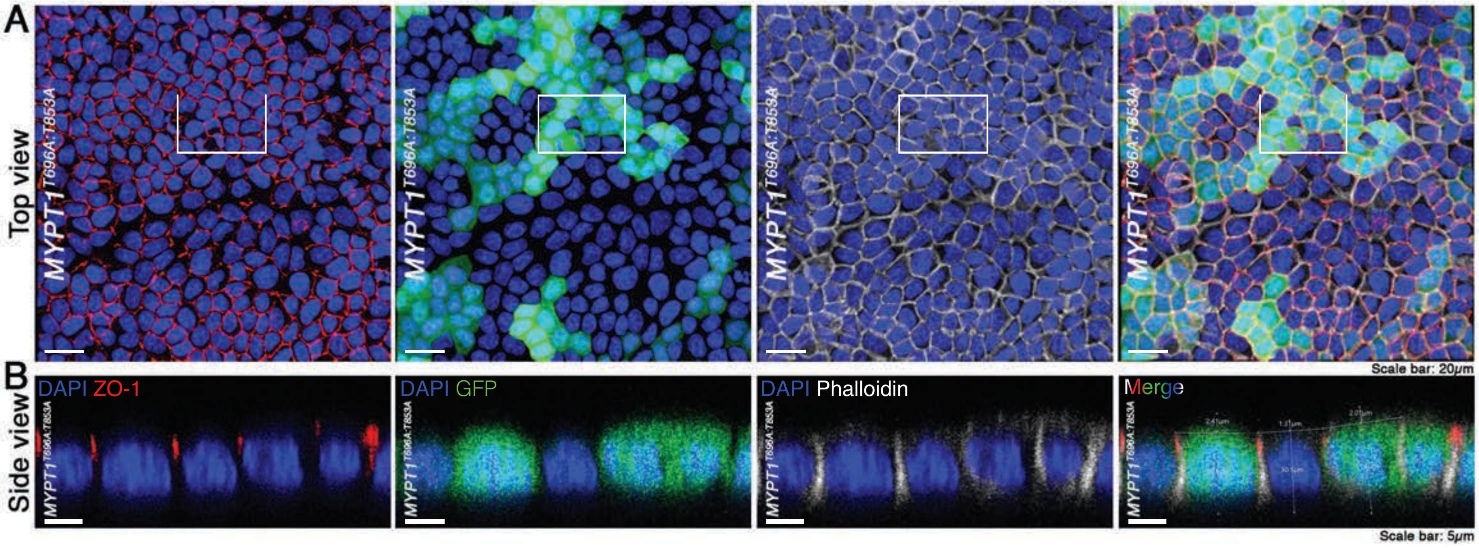

Wu, Herrmann, Hyer, Hua, and Mikawa sFigure 5

sFigure 1. Characterization of MDCK cells at different confluencies.
(A) TEER measurements for MDCK cells grown in culture. MDCK monolayers displayed the highest electrical resistance of 279.6 Ω•cm^2 ± 33.23 Ω•cm^2 s.d. at 45 hours over a 3-day measurement period of the same monolayer. (B) Second TEER experiment for MDCK cells grown in culture. MDCK monolayers displayed the highest electrical resistance of 146.1 kΩ•cm^2 ± 12.33 Ω•cm^2 s.d. at 50 hours over a 3-day measurement period of the same monolayer. (C) Side views of stacked confocal optical sections of MDCK confluent culture or (D) subconfluent culture or (E) overconfluent culture immunostained with ZO-1 (red), β-catenin (green), and filamentous actin dye, phalloidin (grey), and DNA DAPI (blue) counterstain. (F) As in Figure 1 C-E, with added comparison of overconfluent MDCK cell culture. Average macro-height measurements for overconfluent MDCK cells was 8.90 μm ± 1.02 μm s.d., and average micro-height was 1.18 μm ± 0.19 μm s.d. Conditions were statistically compared with a Kruskal-Wallis test ($H(3, 135)=113.7$, $p<0.0001$ for macro-height and micro-height). Unequal variance was confirmed with Levene's test ($p=0.0093$ for macro-height, $p=0.0274$ for micro-height). (E) Standard deviations of macro- and micro-height of confluent and subconfluent monolayers. Flatness threshold indices are indicated with horizontal dashed lines. (Scale bars: 5μm)

sFigure 2. Characterization and quantification of MDCK cells.
(A) Schematic of individual MDCK cell selection from a monolayer. (A') Selected cell in (A) with measurement points (yellow). Measurements were taken by identifying the midpoint (yellow dot) of the apical actin belt using ZO-1 staining (red) and drawing a line (yellow horizontal line). Macro-height was determined by the distance from the midpoint (yellow dot) to the cell base (vertical yellow line). (A") Selected apical domain in (A'). Micro-height was measured as the distance from the midpoint to the peak of phalloidin staining, h, (grey). (B) Steps taken to quantification of macro- and micro-height: 1) Top view of stacked confocal sections of a confluent MDCK monolayer. 2) Side view of the monolayer after a 90º clockwise rotation along the x-axis. 3) X-Z orthogonal plane showing a cross-section of the monolayer for side-view cell measurements. 4) Select individual cell for quantification. 5) Zoom in on the selected cell. 6) Measurement of the apical horizontal line connecting tight junctions via ZO-1. 7) Micro-height measurement as described in (A"). 8) Macro-height measurement as described in (A'). Fluorescent images were thresholded to highlight the top 80% brightness across all channels. (Scale bars: 2, 10μm)

sFigure 3. Confocal optical sections of withaferin A- or nocodazole-treated MDCK cells, immunostained with β-catenin.
(A) and (B) As in Figure 2 (F, G) with β-catenin immunostained rather than cytokeratin or α-tubulin, respectively.

sFigure 4. Transmission electron microscopy (TEM) reveals decreased number of microvilli in ML-7 treated MDCK cells.
(A) Transmission electron microscopy (TEM) for MDCK cells treated with DMSO. (B) As in (A) but treated with 100 μM ML-7. (C) Quantification of microvilli number of DMSO with an average of 35.88 ± 6.13 s.d. and ML-7 treated with an average of 6.75 ± 3.54 s.d. number of microvilli (n=8 cells for each treatment) ($p<0.0001$, $t=11.64$). Treatments were statistically compared with a Student's t-test. Note: ML-7 treated MDCK cells showed a decrease of microvilli number. (Scale bars: 2 μm)

sFigure 5. Transgenic perturbation of myosin II shows maintained GFP(-) micro-height amid surrounding *MYPT1*$^{T696A;T853A}$ mutant MDCK cells

As in Figure 4 B: (A) Top and (B) side views of stacked confocal optical sections of a *MYPT1*$^{T696A;T853A}$ mutant MDCK monolayer transfected with *MYPT1*$^{T696A;T853A}$ plasmid (green) immunostained with ZO-1 (red), and filamentous actin dye, phalloidin (grey), and DNA DAPI (blue) counterstain. Boxed region shows a GFP(-) cell completely surrounded by *MYPT1*$^{T696A;T853A}$ mutant cells. Side views in (B) are cells within the boxed region. Measurement points of GFP- and surrounding *MYPT1*$^{T696A;T853A}$ mutant cells are shown (Scale bars: 20µm, 5µm)
