# Supplementary material for: Steady-state epithelial apical flatness is characterized by MLCK morphodynamics and asynchronous Ca2+ oscillations, but not by underlying ECM geometry
Source: Mol Biol Cell. 2026 Apr 14;37(5):ar45. doi: 10.1091/mbc.E25-12-0583 (PMC13244296; doi:10.1091/mbc.E25-12-0583)
Supplement: Supplementary file 3 [file reviewer_reportE25-12-0583.pdf]

Steady-state epithelial apical flatness is characterized by MLCK morphodynamics and asynchronous Ca2+ oscillations, but not underlying ECM geometry

Hao Wu, Emerson Herrmann, Jeanette Hyer, Lisa Hua, and Takashi Mikawa

Corresponding author(s): Lisa Hua, Sonoma State University

Review Timeline:

Submission Date:	2025-12-12
Editorial Decision:	2026-01-15
Revision Received:	2026-03-16
Accepted:	2026-03-22

Editor-in-Chief: John Aitchison

Transaction Report:

(Note: With the exception of the correction of typographical or spelling errors that could be a source of ambiguity, letters and reports are not edited. The original formatting of letters and referee reports may not be reflected in this compilation.)

RE: Manuscript #E25-12-0583
TITLE: Steady-state epithelial apical flatness is characterized by MLCK morphodynamics and asynchronous Ca2+ oscillations, but not underlying ECM geometry

Dear Dr. Hua:

We have received one review on your article, and in the interests of time and my evaluation, think it is better to proceed with revisions rather than wait longer. I concur with the points of the reviewer, many of which can be addressed by textual changes. However, the points raised in #2 and #5 especially may require some additional experimentation.

Thank you for submitting this exciting work to MBoC and I look forward to seeing your revision.

Sincerely,

Terry Lechler
Monitoring Editor
Molecular Biology of the Cell

Dear Dr. Hua,

The review of your manuscript, referenced above, is now complete. The Monitoring Editor has decided that your manuscript is not acceptable for publication at this time, but may be deemed acceptable after specific revisions are made, as described in the Monitoring Editor's decision letter above and the reviewer comments below.

A reminder: Please do not contact the Monitoring Editor directly regarding your manuscript. If you have any questions regarding the review process or the decision, please contact the MBoC Editorial Office (mboc@ascb.org).

When submitting your revision include a rebuttal letter that details, point-by-point, how the Monitoring Editor's and reviewers' comments have been addressed. (The file type for this letter must be "rebuttal letter"; do not include your response to the Monitoring Editor and reviewers in a "cover letter.") Please bear in mind that your rebuttal letter will be published with your paper if it is accepted, unless you have opted out of publishing the review history.

Authors are allowed 90 days to submit a revision. If this time period is inadequate, please contact us at mboc@ascb.org.

Revised manuscripts are assigned to the original Monitoring Editor whenever possible. However, special circumstances may preclude this. Also, revised manuscripts are often sent out for re-review, usually to the original reviewers when possible. The Monitoring Editor may solicit additional reviews if it is deemed necessary to render a completely informed decision.

In preparing your revised manuscript, please follow the instruction in the Information for Authors (www.molbiolcell.org/info-for-authors). In particular, to prepare for the possible acceptance of your revised manuscript, submit final, publication-quality figures with your revision as described.

To submit the rebuttal letter, revised manuscript, and figures, use this link: Link Not Available

MBoC offers the option to publish your work with immediate open access. Open access can increase the discoverability and usability of your research. If you would like to publish your paper with immediate open access but did not select this option during initial submission, please contact the editorial office at mbc@ascb.org.

Authors of Articles and Brief Communications are also encouraged to create a short video abstract to accompany their article when it is published. These video abstracts, known as Science Sketches, are up to 2 minutes long and will be published on YouTube and then embedded in the article abstract. Science Sketch Editors on the MBoC Editorial Board will guide you as you prepare your video. Information about how to prepare and submit a video abstract is available at www.molbiolcell.org/science-sketches. Please contact mboc@ascb.org if you are interested in creating a Science Sketch.

Sign up for MBoC's new content alerts here https://tinyurl.com/3bcsyk3e

Thank you for submitting your manuscript to MBoC. We look forward to receiving your revised paper.

Sincerely,

Eric Baker
Journal Production Manager
MBoC Editorial Office
mbc@ascb.org

Reviewer #1 (Remarks to the Author):

The article by Wu et al. reports on cytoskeletal involvement in maintaining epithelial flatness in MDCK monolayers. The manuscript is very clearly written, and the figures are well constructed and clear. In addition, the description of statistical methods for each experiment is well done. The paper addresses a fundamental aspect of epithelial biology, how cells maintain flatness of a sheet. Overall, the paper is interesting and mainly descriptive. Below are some improvements that the authors could consider, related to further insights into how flatness is maintained and between topics in the manuscript.

1. The authors first identify maximal epithelial integrity by measuring TEER, then use this to compare cell height at sub-confluent and confluent conditions. The use of macro- and micro-height measurements is a clever way to describe flatness of epithelial cells and tissues. However, the authors could clarify how the bottom is defined for measurements of the whole cell height, since most staining shown seems to be absent from the basal side. The differences in height between subconfluent and confluent conditions is striking. How tightly coupled is TEER to cell height and flatness? For example, beyond 45hours, the TEER drops again to almost subconfluent levels by about 70 hours. What happens to the flatness at this timepoint?

2. The authors demonstrate a role for actin integrity in macro- and micro-height, with some involvement of microtubules in micro-height, but no effect on either in the presence of intermediate filament disruptor. The interpretation of the effect (or lack thereof) of the inhibitors for microtubules and intermediate filaments would be strengthened by staining markers (tubulin, cytokeratin, vimentin) to assess their integrity, similar to the staining with phalloidin that is shown.

3. The results with the MYPT1 mutant that the effect on micro-height is striking and the cell autonomous effect is an interesting finding. As I understand, the confluent epithelial sheet provides some mechanical integrity through cell-cell adhesions. Based on ZO1 and b-catenin staining, these appear to be intact in most of the treatments. Is it possible that some aspects of micro-height are cell autonomous, but requires some level of connectedness? For example, the GFP- cells that border the GFP+ MYPT1-mutant cells, retain normal adhesions to several other cells. Are there examples from the cultures where individual GFP- cells are surrounded by GFP+ cells, such that all of their neighbors are mutant? Do these still retain the "normal" height? Have the authors considered other approaches to validate the cell autonomous effects? For example, uncoupling a cell mechanically from neighbours (e.g. cell ablation of a neighbor) could reveal if there is a requirement for cell adhesion from neighbors and whether the flatness is truly autonomous.

4. The authors could consider additional discussion about the height cells presented and the height of cells from related tissue (e.g. kidney tubule). How does it compare? Furthermore, have the authors considered other epithelial cell types with different geometries (i.e. columnar, cuboidal, squamous)? In some ways, the subconfluent may be more similar to squamous cells and confluent are more similar to cuboidal cells. Are flatness indices expected to be similar for each cell geometry?

5. The involvement of iCa2+ is correlative and the evidence provided does not fully support the interpretation of the data. For example, the conclusions would be strengthened by demonstrating a functional role for iCa2+ in maintaining flatness. Furthermore, if the iCa2+ levels fluctuate, does this imply that myosin activity is also fluctuating to maintain flatness? What might drive these fluctuations and how might this be involved to maintain flatness?

6. The connection between elements of the paper could be further explored (in addition to the iCa2+ mentioned above). For example, the micro-height is greater in sub-confluent versus confluent cultures (Fig. 1D). In confluent cultures, Nocodazole and Cytochalasin both reduce micro-height. Do the inhibitors also impact micro-height in sub-confluent cultures, as it does in confluent (Fig. 3I)? Similarly, do the inhibitors affect cell height changes in the shallow laminin condition? Addressing this would provide more clarity on the connection between elements of the paper.

Molecular Biology of the Cell Reviewer's Response
Title: Steady-state epithelial apical flatness is characterized by MLCK morphodynamics and asynchronous Ca2+ oscillations, but not underlying ECM geometry
Submission ID Manuscript #E25-12-0583
Authors: Wu, Herrmann, Hyer, Hua, and Mikawa

Response to Reviewers
Please find our revised manuscript attached (#E25-12-0583). We would like to thank the reviewer for their helpful comments and constructive criticisms to improve our manuscript. We have incorporated all of their suggestions. We have provided a point-by-point response to the reviewer's critiques:

Reviewer #1
We would like to thank the reviewer for his/her evaluation of our work and constructive critiques and suggestions. We have revised the paper with new data (See Figure 2 E-J, sFigures 1, 3, 5), addition of new and revised text as the reviewer requested. Our response to each specific comment is listed below.

Major points:

Critique #1. *The authors first identify maximal epithelial integrity by measuring TEER, then use this to compare cell height at sub-confluent and confluent conditions. The use of macro- and micro-height measurements is a clever way to describe flatness of epithelial cells and tissues. However, the authors could clarify how the bottom is defined for measurements of the whole cell height, since most staining shown seems to be absent from the basal side. The differences in height between subconfluent and confluent conditions is striking. How tightly coupled is TEER to cell height and flatness? For example, beyond 45hours, the TEER drops again to almost subconfluent levels by about 70 hours. What happens to the flatness at this timepoint?*

Response:

We thank the reviewer for this question and for highlighting the importance of clearly defining how cell height measurements were obtained. We have revised the Materials and Methods section to clarify how the basal side reference point was defined for individual cell height measurements (see revised text/track changes): "*The bottom of the cell was defined by Phalloidin exclusion staining along the apical-basal axis, allowing consistent and reproducible height measurements across conditions*".

We appreciate the reviewer's question regarding the relationship between TEER, cell height, and epithelial flatness over time. We have added new data and analysis (See sFig. 1B, E, F-H). Please note that, for these experiments, we used a new Volt/Ohmmeter, ScopeMeter 190 Series (Fluke) rather than the original Epithelial Volt/Ohm Meter (EVOM; World Precision Instruments). Consequently, TEER measurements were repeated using the new instrument (sFigure 1B), and the updated methodology has been described in the Materials and Methods section (see revised text/track changes).

We have also added new text to the Results see revised text/track changes: "*Our data indicate that TEER and epithelial flatness are closely correlated during the approach to peak confluency. At 45-50 hours, when TEER reaches its maximum, cells exhibit minimal variability in both macro- and micro-height measurements and display a uniform flat apical surface (sFigure 1 A, B, F-H). This suggests that the maximal barrier integrity coincides with maximal epithelial flattening and structural homogeneity of the monolayer. However, beyond peak confluence, the relationship becomes more complex. At 60-75 hours, TEER declines to levels comparable to subconfluent MDCK cultures (sFigure 1 A, B). Despite this drop in barrier function, macro- and micro-height measurements at these later time points, defined as the "overconfluent monolayer", remain closer to those of the confluent monolayer than to subconfluent cells (sFigure 1 E-H).*

While we observe a decrease in macro-height in the overconfluent layer, micro-height remains mostly unchanged as compared to confluent layers (sFigure 1 F, G). However, there is a slight increase in macro-height variability, suggesting a slight loss of epithelial flatness in overconfluent monolayers (sFigure 1 F-H). Although the overall macro-height of the overconfluent monolayer is reduced, it does not revert to the variable, shorter morphology characteristic of subconfluent cells, instead, it displays an intermediate phenotype (sFigure 1 C-H). These findings suggest that TEER and epithelial flatness may be correlated during establishment of the monolayer, but become partially uncoupled in overconfluent conditions."

The decline in TEER after peak confluency may reflect additional processes, such as increased apoptosis, local cell extrusions, junctional remodeling, or mechanical stress accumulation, rather than a simple reversal of cell flattening. Although MDCK monolayers at later time points may exhibit increased apoptosis, the remaining cells appear to maintain their flatness morphology, suggesting the presence of intrinsic or tissue-level mechanisms that preserve epithelial geometry despite local barrier disruptions.

Together, these results indicate that while epithelial flattening contributes to maximal barrier integrity during monolayer formation and establishing, TEER is not determined solely by cell height. Instead, barrier function at later stages likely reflects a combination of junctional organization, cell turnover, and tissue-level remodeling processing, which are beyond the scope of the present study.

Critique #2. *The authors demonstrate a role for actin integrity in macro- and micro-height, with some involvement of microtubules in micro-height, but no effect on either in the presence of intermediate filament disruptor. The interpretation of the effect (or lack thereof) of the inhibitors for microtubules and intermediate filaments would be strengthened by staining markers (tubulin, cytokeratin, vimentin) to assess their integrity, similar to the staining with phalloidin that is shown.*

Response:

We thank the reviewer for this helpful suggestion and have performed new experiments that are consistent with our previous results. See new text/track changes: "*To directly assess the integrity of the cytoskeletal networks following pharmacological perturbation, we performed IF staining for cytokeratin and α-tubulin to visualize intermediate filaments and microtubules, respectively (Figure 2 F-J). These data confirm that treatment with withaferin A perturbs intermediate filament organization (Figure 2 F), while treatment with nocodazole effectively*

disrupts the microtubule network (Figure 2 G). Disruption of intermediate filaments with withaferin A produced changes in macro-, but not micro-height, with s.d. values remaining below the threshold indices used to define loss of flatness (Figure 2 J).

In contrast, inhibition of microtubule polymerization with nocodazole resulted in a significant decrease in apical micro-height (Figure 2 I; $p<0.0001$), while overall macro-height and epithelial flatness were largely preserved (Figure 2 H, J). These results indicate that microtubules contribute selectively to apical, micro-height regulation without substantially affecting overall monolayer flatness.

The data indicate that actin and microtubules contribute to apical micro-height regulation, while actin and intermediate filaments contribute to macro-height regulation. These findings support a model in which actin filaments play a role in regulating overall epithelial flatness."

Critique #3. *The results with the MYPT1 mutant that the effect on micro-height is striking and the cell autonomous effect is an interesting finding. As I understand, the confluent epithelial sheet provides some mechanical integrity through cell-cell adhesions. Based on ZO1 and b-catenin staining, these appear to be intact in most of the treatments. Is it possible that some aspects of micro-height are cell autonomous, but requires some level of connectedness? For example, the GFP- cells that border the GFP+ MYPT1-mutant cells, retain normal adhesions to several other cells. Are there examples from the cultures where individual GFP- cells are surrounded by GFP+ cells, such that all of their neighbors are mutant? Do these still retain the "normal" height? Have the authors considered other approaches to validate the cell autonomous effects? For example, uncoupling a cell mechanically from neighbours (e.g. cell ablation of a neighbor) could reveal if there is a requirement for cell adhesion from neighbors and whether the flatness is truly autonomous.*

Response:

We thank the reviewer for this thoughtful and constructive critique. We agree that the striking effect of the MYPT1 mutant on apical micro-height raises important questions about the degree to which this phenotype is cell autonomous versus influenced by mechanical coupling within the epithelial sheet.

As the reviewer notes, ZO-1 staining indicates that cell to cell junctions remain largely intact in these mosaic monolayers, suggesting that overall epithelial cohesion is preserved. We have clarified this point in the Results section and added representative examples to new sFig 4. See new text/track changes: "*GFP(-) cells adjacent to and surrounded by GFP(+) MYPT1$^{T696A;T853A}$-mutant cells retain junctional contacts with multiple neighboring mutant cells (sFigure 5). In cases where GFP(-) cells were bordered by multiple GFP(+) MYPT1$^{T696A;T853A}$-mutant neighbors, these cells still maintained normal micro-height under these conditions (sFigure 5), supporting the idea that the MYPT1-dependent micro-height phenotype is primarily cell autonomous. The available data suggest that the increased micro-height phenotype does not propagate to neighboring GFP(-) cells and is primarily cell-autonomous.*"

We appreciate the reviewer's suggestion of mechanically uncoupling cells by laser ablation as a potential approach to directly test the requirement for neighbor connectivity. While these experiments are technically challenging in confluent MDCK monolayers and may

introduce confounding cytotoxic effects (Ibrahim et al., 2024; Ganjali et al., 2018; Liu et al., 2019), we agree that they represent valuable future directions. We have therefore modified the Discussion text/track changes to state: "*Micro-height regulation is largely cell autonomous under the conditions tested, while we acknowledge a basal level of epithelial connectivity may still contribute to overall tissue mechanics. Together, our data support a model in which MYPT1-dependent regulation of apical micro-height operates in a predominantly cell-autonomous manner, even within a mechanically integrated epithelial sheet.*"

Critique #4. The authors could consider additional discussion about the height cells presented and the height of cells from related tissue (e.g. kidney tubule). How does it compare? Furthermore, have the authors considered other epithelial cell types with different geometries (i.e. columnar, cuboidal, squamous)? In some ways, the subconfluent may be more similar to squamous cells and confluent are more similar to cuboidal cells. Are flatness indices expected to be similar for each cell geometry?

Response:

We agree that placing our cell height measurements into the context of epithelial geometry in vivo strengthens the interpretation of our findings. We have now added new text/track changes in the discussion comparing the measured heights of MDCK monolayers to reported dimensions of renal tubular epithelial cells: "*MDCK cells are derived from canine kidney distal tubule epithelium, which in vivo exhibits a predominantly cuboidal morphology (Cho et al., 1989). The cultured renal epithelial cell heights we measure under confluent conditions are consistent with a cuboidal organization rather than a squamous phenotype (Cai and Mostov, 2012). In contrast, subconfluent MDCK cells display increased variability in height and surface contour, which may resemble a less organized or partially spread epithelial state rather than a physiologically defined squamous epithelium (Dukes et al., 2011, Chmiel & Gardel, 2022).*"

We also appreciate the reviewer's broader question regarding epithelial geometry. We have clarified this conceptual distinction in the Discussion and now emphasize that (See new text/track changes: "*Epithelia* in vivo *exhibit a wide range of morphology including squamous (such as endothelium), cuboidal (such as renal tubules), and columnar (such as intestinal epithelium). Each of these epithelia are associated with distinct mechanical and functional environments and processes (Bartle et al., 2018). We do not necessarily expect absolute cell height or flatness indices to be identical across these epithelial geometries. Rather, we propose that our flatness indices are best interpreted as measures of monolayer uniformity rather than absolute cell height. Flatness reflects how consistently cells maintain a shared apical surface within a given epithelial environment, independent of whether the tissue is squamous, cuboidal, or columnar. The flatness index may be a geometry-independent metric of epithelial surface uniformity. Future studies comparing epithelial types with defined in vivo geometries would further test how broadly this metric applies across tissues.*"

Critique #5. *The involvement of iCa2+ is correlative and the evidence provided does not fully support the interpretation of the data. For example, the conclusions would be strengthened by demonstrating a functional role for iCa2+ in maintaining flatness. Furthermore, if the iCa2+ levels*

fluctuate, does this imply that myosin activity is also fluctuating to maintain flatness? What might drive these fluctuations and how might this be involved to maintain flatness?

Response:

We thank the reviewer for this important comment regarding the role of iCa^{2+}. We agree that the current data do not establish a direct functional requirement for iCa^{2+} in maintaining epithelial flatness. Our intent was not to position Ca2+ signaling as a mechanistic driver of flatness, but rather to report the presence of asynchronous, cell-autonomous iCa^{2+} oscillations occurring within confluent epithelial monolayers.

We have revised the manuscript to clarify that the Ca2+ imaging findings are presented as a parallel observation, not as definitive mechanistic evidence. The revised text positions the Ca2+ data as hypothesis-generating and complementary, while keeping the primary mechanistic focus on actomyosin-mediated height regulation. We have also removed language implying calcium dependence from the abstract, and results, and have toned down the Discussion to avoid over-interpretation. See new revised abstract: *"Finally, Ca^{2+} imaging reveals an asynchronous calcium flux across a confluent epithelium."*

We now explicitly state this limitation in the Discussion: See text/track changes: "*The conclusions of the study, that actomyosin contractility regulates micro- and macro-height are supported independently of the iCa^{2+} data. The iCa^{2+} measurements are included to highlight an additional layer of dynamic intracellular signaling that may be compatible with the observed cell-autonomous regulation of apical height. With respect to whether iCa^{2+} fluctuations imply corresponding myosin fluctuations, we acknowledge that our current temporal and spatial resolution does not allow direct coupling of individual iCa^{2+} transients to myosin activity. Demonstrating such causality would require simultaneous high-resolution biosensors for calcium and myosin activity.",* which is beyond the scope of this study.

Critique #6. The connection between elements of the paper could be further explored (in addition to the iCa2+ mentioned above). For example, the micro-height is greater in sub-confluent versus confluent cultures (Fig. 1D). In confluent cultures, Nocodazole and Cytochalasin both reduce micro-height. Do the inhibitors also impact micro-height in sub-confluent cultures, as it does in confluent (Fig. 3I)? Similarly, do the inhibitors affect cell height changes in the shallow laminin condition? Addressing this would provide more clarity on the connection between elements of the paper.

Response:

We would like to thank the reviewer for suggesting a better integration of the different elements of the paper. We have therefore moved Fig 2 to Fig 6 and the corresponding text to the end of the Results. This revised order will better guide the conceptual framework linking subconfluent, confluent, and altered-basement membrane conditions via laminin patch.

Our primary objective was to define mechanisms regulating epithelial flatness under conditions in which a mechanically integrated confluent monolayer is established. For this reason, the pharmacological perturbation experiments shown in Figure 2 were performed under confluent

conditions, where micro-height regulation reflects coordinated epithelial architecture rather than isolated single-cell behavior.

In subconfluent cultures, cells exhibit greater heterogeneity in spreading, polarity, and junctional organization, and micro-height variability is increased (Figure 1D). Under these conditions, cell shape may be influenced by substrate adhesion and incomplete cell to cell coupling (Abu Taha & Schnittler, 2014; Cammarota et al., 2024; Dawney et al., 2023; Revel et al., 1971). As a result, pharmacological perturbations of actin (Cytochalasin D) or microtubules (nocodazole) in subconfluent cultures primarily affect overall cell morphology and spreading, making interpretation of micro-height changes less directly comparable to those observed in confluent monolayers. For clarity and mechanistic focus, we therefore restricted quantitative inhibitor analysis to confluent MDCK monolayers only (Figure 2).

Similarly, in shallow laminin patch (Type 2) conditions, there may be changes in adhesion and cell-matrix interactions that alter overall cell architecture (Figure 6). In that context, height regulation reflects altered substrate mechanics via basal modulation rather than cytoskeletal components within a stable epithelial sheet (Töpfer, 2023; Janmey et al., 2020; Yang et al., 2017; Breuls et al., 2008; Xie et al., 2018). We have now clarified this distinction in the Discussion (See new text/track changes) emphasizing that *"our inhibitor experiments were designed to interrogate cytoskeletal fibers specifically within an established MDCK epithelial monolayer*."

We agree with the reviewer that examining all conditions in parallel would be informative. However, such additional experiments and subsequent analyses would require careful redefinition of flatness metrics across different mechanical states and are beyond the scope of the current study.

RE: Manuscript #E25-12-0583R
TITLE: "Steady-state epithelial apical flatness is characterized by MLCK morphodynamics and asynchronous Ca2+ oscillations, but not underlying ECM geometry"

Dear Dr. Hua:

I am pleased to accept your manuscript for publication in Molecular Biology of the Cell. Congratulations, and thank you for sending this work to MBoC.

Sincerely,
Terry Lechler
Monitoring Editor
Molecular Biology of the Cell

Dear Dr. Hua:

Congratulations on the acceptance of your manuscript! Thank you for publishing your work in Molecular Biology of the Cell (MBoC).

Within 10 days, an unedited PDF of your manuscript will be published on MBoC in Press, an early-release journal version. The date your manuscript appears on this site is the official publication date.

Your copyedited and typeset manuscript will be scheduled for publication in the next available issue of MBoC. Once your paper is ready for review, our production team will notify you. In the summer of 2024, our production provider will introduce an online proofing solution, which will streamline the process of checking and validating author changes. This will result in improved typesetting quality, enhanced digital content, and a faster overall process.

We invite you to submit images related to your accepted manuscript to be considered for the journal cover.
Cover images must contain color, TIF Format
Size image at 7.4" wide x 5.85" deep
RGB and CMYK color (please submit two separate files, one in each format)
300 dpi resolution

Please also send a 1-paragraph description of the image that a general audience could understand. Include with the caption the name and institution of the individual(s) the image should be attributed to. Please provide this description in a Word file. You can send the file via email or by your favorite file transfer service to mboc@ascb.org.

Authors of Articles and Brief Communications are also encouraged to create a short video abstract to accompany their article when it is published. These video abstracts, known as Science Sketches, are up to 2 minutes long and will be published on YouTube and then embedded in the article abstract. Science Sketch Editors on the MBoC Editorial Board will guide you as you prepare your video. Information about how to prepare and submit a video abstract is available at www.molbiolcell.org/science-sketches. Please contact mboc@ascb.org if you are interested in creating a Science Sketch.

We look forward to publishing your paper in MBoC.
Sign up for MBoC's new content alerts here https://tinyurl.com/3bcsyk3e

Sincerely,

Eric Baker
Journal Production Manager
MBoC Editorial Office
mbc@ascb.org
